# An Expert Consensus Statement on Biomarkers of Aging for Use in Intervention Studies

**DOI:** 10.1093/gerona/glae297

**Published:** 2024-12-21

**Authors:** Giorgia Perri, Chloe French, César Agostinis-Sobrinho, Atul Anand, Radiana Dhewayani Antarianto, Yasumichi Arai, Joseph A Baur, Omar Cauli, Morgane Clivaz-Duc, Giuseppe Colloca, Constantinos Demetriades, Chiara de Lucia, Giorgio Di Gessa, Breno S Diniz, Catherine L Dotchin, Gillian Eaglestone, Bradley T Elliott, Mark A Espeland, Luigi Ferrucci, James Fisher, Dimitris K Grammatopoulos, Novi S Hardiany, Zaki Hassan-Smith, Waylon J Hastings, Swati Jain, Peter K Joshi, Theodora Katsila, Graham J Kemp, Omid A Khaiyat, Dudley W Lamming, Jose Lara Gallegos, Frank Madeo, Andrea B Maier, Carmen Martin-Ruiz, Ian J Martins, John C Mathers, Lewis R Mattin, Reshma A Merchant, Alexey Moskalev, Ognian Neytchev, Mary Ni Lochlainn, Claire M Owen, Stuart M Phillips, Jedd Pratt, Konstantinos Prokopidis, Nicholas J W Rattray, María Rúa-Alonso, Lutz Schomburg, David Scott, Sangeetha Shyam, Elina Sillanpää, Michelle M C Tan, Ruth Teh, Stephanie W Tobin, Carolina J Vila-Chã, Luigi Vorluni, Daniela Weber, Ailsa Welch, Daisy Wilson, Thomas Wilson, Tongbiao Zhao, Elena Philippou, Viktor I Korolchuk, Oliver M Shannon

**Affiliations:** Human Nutrition & Exercise Research Centre, Centre for Healthier Lives, Population Health Sciences Institute, Newcastle University, Newcastle Upon Tyne, UK; School of Health Sciences, University of Manchester, Manchester, UK; Sport Physical Activity and Health Research & Innovation Center (SPRINT), Guarda, Portugal; Health Research and Innovation Science Centre, Klaipeda University, Klaipeda, Lithuania; Centre for Cardiovascular Science, University of Edinburgh, Edinburgh, UK; Department of Histology, Faculty of Medicine, Universitas Indonesia, Jakarta, Indonesia; Stem Cell and Tissue Engineering, Indonesian Medical Education and Research Institute (IMERI), Faculty of Medicine, Universitas Indonesia, Jakarta, Indonesia; Center for Supercentenarian Medical Research, Keio University School of Medicine, Tokyo, Japan; Department of Physiology and Institute for Diabetes, Obesity, and Metabolism, Perelman School of Medicine, University of Pennsylvania, Philadelphia, Pennsylvania, USA; Department of Nursing, University of Valencia, Valencia, Spain; Chair of Active Ageing, University of Valencia, Valencia, Spain; CareConnex, Sierre, Valais, Switzerland; Dipartimento di Diagnostica per Immagini, Radioterapia Oncologica Ed Ematologia, Fondazione Policlinico Universitario Agostino Gemelli IRCCS, Rome, Italy; Max Planck Institute for Biology of Ageing (MPI-AGE), Cologne, Germany; Cologne Excellence Cluster on Cellular Stress Responses in Aging-Associated Diseases (CECAD), University of Cologne, Cologne, Germany; Centre for Age-Related Medicine, Stavanger University Hospital, Stavanger, Norway; Department of Basic and Clinical Neuroscience, Institute of Psychiatry, Psychology & Neuroscience, King’s College London, London, UK; Department of Epidemiology & Public Health, University College London, London, UK; UConn Center on Aging & Department of Psychiatry, University of Connecticut Medical School, Farmington, Connecticut, USA; Population Health Sciences Institute, Newcastle University, Newcastle upon Tyne, UK; Northumbria Healthcare NHS Foundation Trust, North Shields, UK; Institute for Lifecourse Development, School of Health Sciences, University of Greenwich, London, UK; Ageing Biology & Age Related Diseases, School of Life Sciences, University of Westminster, London, UK; Department of Internal Medicine, Wake Forest University School of Medicine, Winston-Salem, North Carolina, USA; Translational Gerontology Branch, Biomedical Research Center, National Institute on Aging, National Institutes of Health, Baltimore, Maryland, USA; School of Medicine, Newcastle University, Newcastle upon Tyne, UK; Biomedical Sciences, Warwick Medical School, University of Warwick, Coventry, UK; Institute of Precision Diagnostics and Translational Medicine, Pathology, University Hospital Coventry and Warwickshire NHS Trust, Coventry, West Midlands, UK; Department of Biochemistry & Molecular Biology, Faculty of Medicine, Universitas Indonesia, Jakarta, Indonesia; Institute for Metabolism and Systems Research, University of Birmingham, Birmingham, UK; Department of Endocrinology, University Hospitals Birmingham NHS Foundation Trust, Birmingham, UK; Department of Nutrition, Texas A&M University, College Station, Texas, USA; World Public Health Nutrition Association, Peacehaven, UK; Humanity Inc, Humanity, Boston, Massachusetts, USA; Centre for Global Health Research, Usher Institute, University of Edinburgh, Edinburgh, UK; Institute of Chemical Biology, Laboratory of Biomarker Discovery & Translational Research, National Hellenic Research Foundation, Athens, Greece; Institute of Life Course and Medical Sciences, Faculty of Health and Life Sciences, University of Liverpool, Liverpool, UK; School of Health and Sport Sciences, Musculoskeletal Health & Rehabilitation, Liverpool Hope University, Liverpool, UK; Department of Medicine, University of Wisconsin-Madison, Madison, Wisconsin, USA; Department of Applied Sciences, Faculty of Health and Life Sciences, Northumbria University, Newcastle upon Tyne, UK; NUTRAN, Applied Sciences, Northumbria University, Newcastle upon Tyne, UK; Institute of Molecular Biosciences, NAWI Graz, University of Graz, Graz, Austria; Field of Excellence BioHealth, University of Graz, Graz, Austria; Healthy Longevity Translational Research Program, Yong Loo Lin School of Medicine, National University of Singapore, Singapore, Singapore; Centre for Healthy Longevity, @AgeSingapore, National University Health System, Singapore, Singapore; BioScreening Core Facility, Translational and Clinical Research Institute, Faculty of Medical Sciences, Newcastle University, Newcastle upon Tyne, UK; Sarich Neuroscience Research Institute, Edith Cowan University, Nedlands, Western Australia, Australia; Human Nutrition & Exercise Research Centre, Centre for Healthier Lives, Population Health Sciences Institute, Newcastle University, Newcastle Upon Tyne, UK; Ageing Biology & Age Related Diseases, School of Life Sciences, University of Westminster, London, UK; Department of Medicine, Yong Loo Lin School of Medicine, National University of Singapore, Singapore, Singapore; Institute of Biogerontology, Lobachevsky State University of Nizhny Novgorod, Nizhny Novgorod, Russia; Research Clinical Center of Gerontology of the National Research Medical University, Moscow, Russia; College of Medical, Veterinary & Life Sciences, School of Molecular Biosciences, University of Glasgow, Glasgow, UK; Department of Twin Research and Genetic Epidemiology, King’s College London, London, UK; Population Health Sciences Institute, Newcastle University, Newcastle upon Tyne, UK; Department of Kinesiology, McMaster University, Hamilton, Ontario, Canada; Department of Sport and Exercise Sciences, Manchester Metropolitan University Institute of Sport, Manchester, UK; Department of Musculoskeletal and Ageing Science, Institute of Life Course and Medical Sciences, University of Liverpool, Liverpool, UK; Strathclyde Institute of Pharmacy and Biomedical Sciences, Faculty of Science, University of Strathclyde, Glasgow, UK; Sport Physical Activity and Health Research & Innovation Center (SPRINT), Guarda, Portugal; Performance and Health Group, Faculty of Sports Sciences and Physical Education, Department of Physical Education and Sports, University of A Coruna, A Coruña, Spain; Institute for Experimental Endocrinology, Max Rubner Center, Charité University Berlin, Berlin, Germany; Institute for Physical Activity and Nutrition (IPAN), School of Exercise and Nutrition Sciences, Deakin University, Geelong, Australia; Faculty of Medicine, Nursing and Health Sciences, School of Clinical Sciences at Monash Health, Monash University, Clayton, Australia; Institut d’Investigació Sanitària Pere Virgili (IISPV), Food, Nutrition, Development and Mental Health (ANUT-DSM) Research Group , Rovira i Virgili University, Reus, Spain; Centro de Investigación Biomédica en Red Fisiopatología de la Obesidad y la Nutrición (CIBEROBN), Institute of Health Carlos III, Madrid, Spain; Gerontology Research Center, Faculty of Sport and Health Sciences, University of Jyväskylä, Jyväskylän yliopisto, Finland; Wellbeing Services County of Central Finland, Jyväskylä, Finland; Population Health Sciences Institute, Newcastle University, Newcastle upon Tyne, UK; Department of Health Service and Population Research, Institute of Psychiatry, Psychology & Neuroscience (IoPPN), King’s College London, London, UK; Department of General Practice and Primary Health Care, Faculty of Medicine and Health Sciences, University of Auckland, Auckland, New Zealand; Trent Centre for Aging & Society, Trent University, Peterborough, Ontario, Canada; Sport Physical Activity and Health Research & Innovation Center (SPRINT), Guarda, Portugal; Independent Researcher, Human Physiology and Integrative Medicine, London, UK; Department of Molecular Toxicology, German Institute of Human Nutrition Potsdam-Rehbrücke (DIfE), Nuthetal, Germany; Centre for Population Health Research, Faculty of Health, University of East Anglia, Norwich, UK; Institute of Inflammation and Ageing, College of Medical and Dental Sciences, University of Birmingham, Birmingham, UK; Department of Life Sciences, Aberystwyth University, Ceredigion, UK; State Key Laboratory of Stem Cell and Reproductive Biology, Institute for Stem Cell and Regeneration, Institute of Zoology, Chinese Academy of Sciences, Beijing, China; Department of Life Sciences, School of Life and Health Sciences, University of Nicosia, Nicosia, Cyprus; Department of Nutritional Sciences, King’s College London, London, UK; Faculty of Medical Sciences, Biosciences Institute, Newcastle University, Newcastle upon Tyne, UK; Human Nutrition & Exercise Research Centre, Centre for Healthier Lives, Population Health Sciences Institute, Newcastle University, Newcastle Upon Tyne, UK; (Biological Sciences Section)

**Keywords:** Consensus, Delphi method, Longevity

## Abstract

Biomarkers of aging serve as important outcome measures in longevity-promoting interventions. However, there is limited consensus on which specific biomarkers are most appropriate for human intervention studies. This work aimed to address this need by establishing an expert consensus on biomarkers of aging for use in intervention studies via the Delphi method.

A 3-round Delphi study was conducted using an online platform. In Round 1, expert panel members provided suggestions for candidate biomarkers of aging. In Rounds 2 and 3, they voted on 500 initial statements (yes/no) relating to 20 biomarkers of aging. Panel members could abstain from voting on biomarkers outside their expertise. Consensus was reached when there was ≥70% agreement on a statement/biomarker.

Of the 460 international panel members invited to participate, 116 completed Round 1, 87 completed Round 2, and 60 completed Round 3. Across the 3 rounds, 14 biomarkers met consensus that spanned physiological (eg, insulin-like growth factor 1, growth-differentiating factor-15), inflammatory (eg, high sensitivity C-reactive protein, interleukin-6), functional (eg, muscle mass, muscle strength, hand grip strength, Timed-Up-and-Go, gait speed, standing balance test, frailty index, cognitive health, blood pressure), and epigenetic (eg, DNA methylation/epigenetic clocks) domains.

Expert consensus identified 14 potential biomarkers of aging which may be used as outcome measures in intervention studies. Future aging research should identify which combination of these biomarkers has the greatest utility.

## Graphical Abstract

**Figure:**
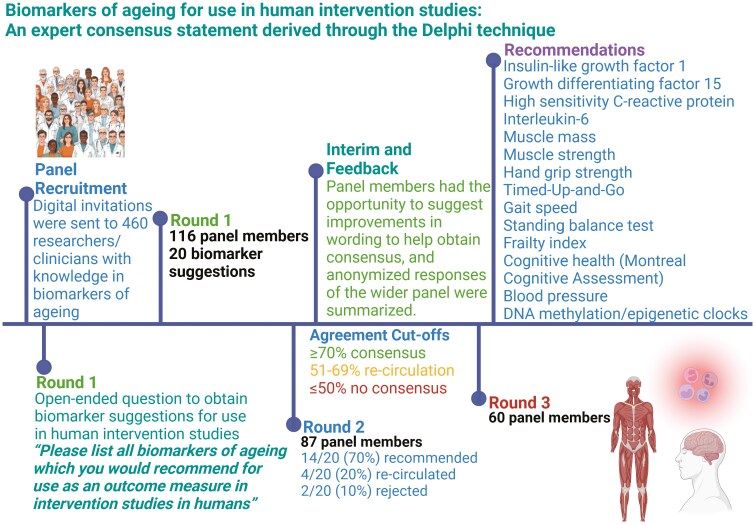
A graphical abstract to depict the methodology of results of this Delphi study.

By 2030, it is estimated that 1 in 6 people globally will be aged over 60 years. Meanwhile, the number of adults aged >80 years is predicted to triple between 2020 and 2050, reaching 426 million (1). Aging is associated with poorer health, reduced physiological reserve, and lower survival rates due to the accumulation of molecular and cellular damage and is generally accompanied by an increased risk of acute and chronic conditions. However, there is heterogeneity in aging trajectories between individuals due to differences in genetic background, as well as lifestyle and environmental exposures. Understanding the underlying mechanisms of the aging process and identifying strategies to improve the aging trajectory is a major research and public health priority.

Biomarkers of aging can be defined as “*quantitative parameters of an organism that, either alone or in a composite, predict biological age and ideally its changes in response to age-related interventions*” (2,3). Biomarkers of aging can be used to understand and monitor the aging process and can help strengthen understanding of the factors responsible for inter-individual differences in aging.

Previous research (3–12) has provided guidance on the features of an appropriate biomarker of aging, including: (1) relevance to aging; (2) minimally invasive and reliable measurement; (3) prediction of functional/biological aspects of aging, for example, mortality, better than chronological age; (4) responsiveness to longevity-promoting interventions; (5) being quantifiable without subjective assessment; (6) results generated by an assay that is adaptable to routine clinical practice and has a timely turnaround (ie, in days vs weeks); (7) high sensitivity and specificity; (8) detectability using easily accessible specimens; and (9) the ability to monitor aging independent of the effect of disease processes (13–15). Although research in this field is growing, with established biomarkers of aging consortia (3,7,16) contributing to this area, there is currently no international consensus on the most appropriate biomarkers currently available for use as outcomes in human intervention studies.

Therefore, the primary aim of this study was to establish a multi-national consensus on appropriate biomarkers of aging for use in human intervention studies. The secondary aim was to provide insight into the suitability of the recommended biomarkers for use in different research settings (which has been highlighted as a research priority in recent reviews) (3,17). It is anticipated that the findings from this study, in conjunction with the results of longitudinal studies focusing on biomarkers, may help inform the design of future intervention studies investigating the aging process.

## Method

### Delphi Method

The Delphi method is a flexible, scientific approach for providing expert consensus on any given topic, especially when empirical evidence is limited or controversial (18,19). Although there is no universally accepted framework for conducting a Delphi method, some key features include: (1) anonymity of panel members, allowing for the removal of bias associated with opinions; (2) controlled feedback; (3) viewing of the overall group response; and (4) adoption of an iterative approach (usually 3 rounds) (19,20). For this study, data were collected and managed using REDCap electronic data capture tools (21,22). Data collection took place between October 2023 and February 2024. Following each round, responses were analyzed by the research team (GP, CF, EP, VIK, OMS) and feedback was provided to the panel members after anonymization.

### Selection and Recruitment of Expert Panel Members

Diversity in the demographics and professional experience of panel members is a preferred criterion of Delphi methods (19). Therefore, expert panel members (researchers and clinicians) from a range of aging-related disciplines were invited to participate based on their expertise or experience in aging and/or biomarker research. Panel members were required to have ≥1 first or last author publication involving biomarkers of aging and/or be an applied practitioner/clinician with practical experience of working with older adults (65+ years) and using biomarkers to predict future health/longevity. In addition, panel members were required to be English-speaking and aged ≥18 years. Invitations were sent out to pre-identified researchers via emails and ResearchGate (https://www.researchgate.net/). Invitations were also distributed via learned societies and research groups/networks associated with aging and biomarker research to ensure a broad coverage of researchers in the field of aging (Supplementary Table 1).

Panel members were asked to share their age, area of expertise, associated research group, career stage, career location, and clinician status. During the recruitment process (which was conducted parallel to Round 1), purposive sampling was used to maximize the diversity of expertise and global representation in the panel. If specialist areas were missing, attempts were made to recruit panel members with expertise in these areas. A dropout rate of 20% was anticipated over the 3 rounds (23,24) and, considering the breadth of this research topic, we aimed to recruit >50 panel members to capture a variety of opinions from different disciplines. It is of note that there are currently no guidelines on selecting a sample size for multi-disciplinary research using the Delphi approach (23) although ~20 panel members have been deemed sufficient for homogeneous samples (25). Finally, panel members who completed the 3 rounds had the opportunity to be involved in the manuscript as co-authors.

### Ethics

Newcastle University granted ethical approval for this study (35295/2023). Panel members provided informed consent using REDCap before commencing Round 1.

### Pilot Testing

Prior to Round 1, different options for the open-ended question were pilot tested on native and non-native English speakers. Ten researchers at Newcastle University (UK) were asked to provide feedback on comprehensibility and select their preferred wording for the question. The highest scoring question was selected for use in Round 1.

### Round 1

Consenting panel members were directed to Round 1 of the Delphi study automatically. Panel members were provided with a definition of “biomarkers of aging” (13) and “aging” (26) as a guide to ensure consistent interpretation of the open-ended question. Panel members were presented with the following open-ended question: “*Please list all biomarkers of ageing which you would recommend for use as an outcome measure in intervention studies in humans*.” This was followed by a free text box for responses. Presenting an open-ended question was considered preferable over proposing a list of biomarkers by the research team to minimize bias introduced by researcher opinions. Panel members were provided with 6 weeks to complete Round 1. Automated reminder emails were sent weekly to maximize response rate.

### Round 2

The answers from Round 1 were collated and biomarkers with similar constructs were manually grouped and refined. Biomarkers were selected for Round 2 if they were suggested 10 or more times. The use of this threshold for selecting biomarkers for Round 2 was based around a practical decision to minimize panel member burden by avoiding the inclusion of potentially irrelevant biomarkers. An invitation to Round 2 was sent out to panel members who completed Round 1. In this round, panel members were asked to appraise only those biomarkers which they believed to be within their area of expertise. Panel members were asked whether they would recommend the biomarker for use in intervention studies and were also presented with an additional 25 statements regarding the suitability of the biomarker for use in interventions (Table 1). The 25 statements were based on previous literature associated with biomarkers of aging (13–15,27). Responses for each biomarker were binary (yes or no) with the option to skip a question if a panel member was unsure, did not have expertise in that biomarker, or felt there was not enough evidence to answer.

**Table 1.** A List of Statements (Referred to Here as “Statements”) Provided to Panel Members for Each Biomarker in Round 2 and Round 3 (When Required)

Statements
Do you have expertise in this biomarker?
Would you recommend this as an appropriate biomarker of aging for use in intervention studies?
Statements
Suitable as an outcome for acute intervention studies? Suitable as an outcome for short-term intervention (<3 months) studies? Suitable as an outcome for medium-term intervention (3–6 months) studies? Suitable as an outcome for long-term intervention (≥6 months) studies? Suitable for field settings? Suitable for cognitively impaired participants? Suitable for frail participants? Does the act of measuring this biomarker accelerate aging? Is it clinically validated (ie, has it been validated for use in clinical settings against set clinical standards)? Is it mechanistically validated (ie, does the biomarker reflect underlying cellular and molecular mechanisms of aging)? Is it generalizable (ie, does the biomarker function across different applications, eg, cell type, organ, system, human populations)? Is it precise (ie, repeatable, and reproducible)? Is it reliable (ie, repeatable with minimal technical variability)? Are sampling and source materials easily obtained including collection, storage, and processing? Are complex models or software required for interpretation? Is it sensitive? Is it specific? Can it be blinded to participants? Can it be blinded to researchers? Can it be blinded to data analysts? Does it predict functional aspects of aging better than chronological aging? Is it responsive (ie, does it respond to accelerated or decelerated aging)? Is this biomarker of...
Minimal burden
Moderate burden
Burdensome
24. Is this biomarker...
Noninvasive
Moderately invasive
Invasive
25. Is this biomarker of...
Minimal financial cost (<$10/participant)
Low financial cost ($10–50/participant)
Moderate financial cost ($51–100/participant)
High financial cost ($101–1000+/participant)

A threshold was set a priori to determine the level of agreement required for consensus. Consensus was determined as 70% or more of panel members agreeing on a statement. If a biomarker reached this threshold, it was accepted as having reached consensus and removed from further voting in Round 3. All statements for which there was less than 50% agreement between panel members were removed from further voting due to perceived redundancy. Statements that did not reach consensus but for which there was moderate agreement between panel members (51%–69%) were reevaluated in Round 3. These thresholds were selected based on previous Delphi methods, including those exploring biomarkers (28–30). When calculating the percentage of responses for each statement, the denominator was based on the number of panel members who reported expertise for that particular biomarker. Panel members were provided with 5 weeks to complete Round 2 and automated reminder emails were sent weekly.

### Interim Round

Prior to Round 3, panel members were given the opportunity to share any feedback on the content of Round 2. A text box was provided for suggestions (eg, to improve the wording of the statements to better reflect the views of the panel) that may increase the likelihood of achieving consensus in Round 3. Panel members were also able to see the list of biomarkers that had not yet reached consensus and would be reevaluated in Round 3. Panel members were provided with 1 week to complete this Round.

### Round 3

Round 3 was the final round of the survey in which all statements (biomarker recommendations and statements) that had not yet reached consensus were reevaluated. In this round, the results from Round 2 were shared anonymously with the panel members who completed Rounds 1 and 2. Panel members were able to view their previous responses, alongside a summary of the overall group voting from Round 2. They then had the option to keep or alter their responses in consideration of the responses from the wider panel. Panel members were asked to appraise all statements which had not yet reached consensus. Panel members were also asked an additional question on whether they would recommend composite biomarkers for use in intervention studies. Panel members were provided with 3 weeks to complete Round 3 and automated reminder emails were sent weekly.

### Data Analysis

Counts and percentages of responses for each statement and biomarker were calculated for each round on Microsoft Excel. Descriptive analyses were performed using SPSS (version 26) (SPSS Inc., Chicago, IL). Frequencies of responses (yes/no) determined the level of agreement in each round and dictated which biomarkers would be reevaluated in Round 3. Chi-square tests were used to compare the differences in characteristics in panel members across the 3 rounds to evaluate risk of selection bias.

## Results

**Figure 1.**
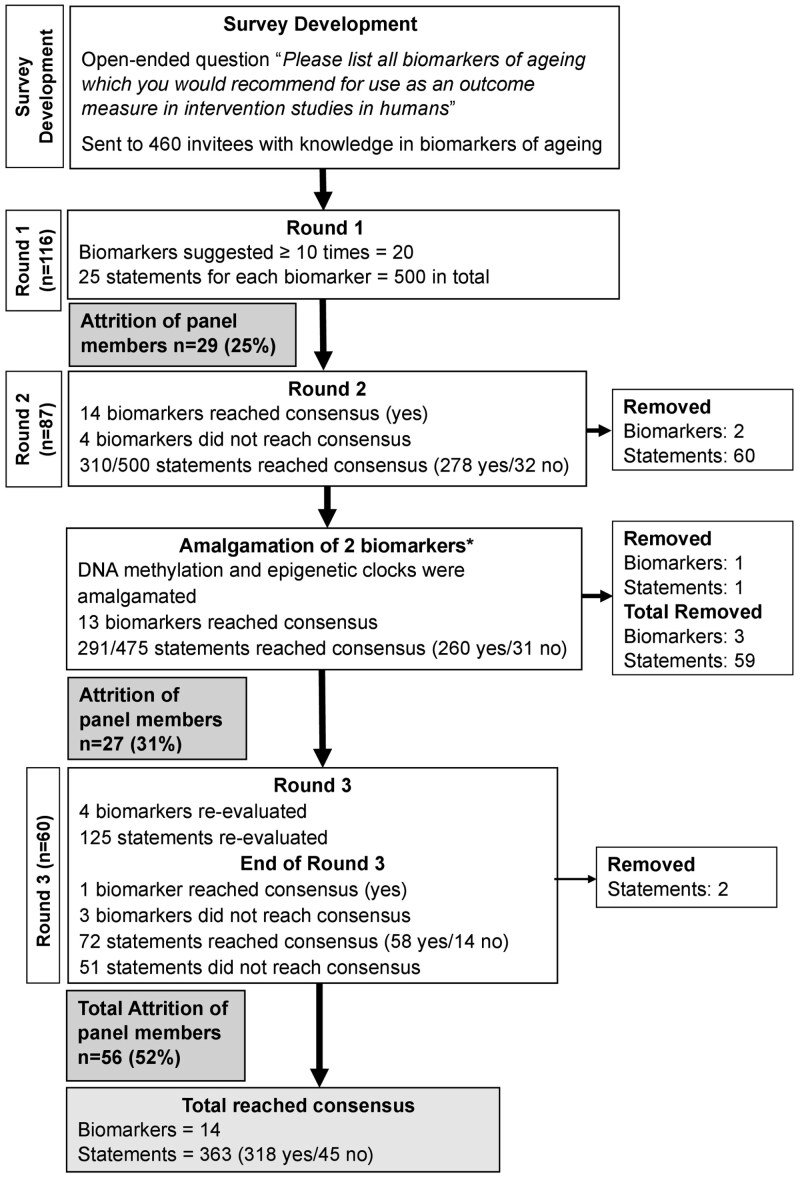
Flow diagram of the Delphi process and results with indications of biomarkers and statements reaching consensus across each round. Numbers in parentheses indicate the numbers of statements reaching consensus (yes or no). *In Round 2, two biomarkers were amalgamated thus reducing the total number of statements from 500 to 475 and resulting in a total of 13 accepted (yes) by the end of Round 2, 125 undecided, and 59 removed biomarkers.

In total, 460 invitations were sent to potential panel members. Of these, 150 panel members (32% response rate) consented to participate, of whom 116 (77%) completed Round 1. Eighty-seven panel members (75%) completed Round 2 and 60 (69%) completed all 3 rounds. There were more non-clinicians (73%) than clinicians, with a slightly larger percentage of panel members in senior roles (self-defined long-term career stages with higher levels of autonomy, responsibility, or leadership) (42%) and the majority of these resided in Europe (65%) (Supplementary Table 2, Supplementary Figure 1). There were no differences in characteristics of the panel members across the rounds (clinician vs non-clinician, career stage, country of location, *p* > .05, data not shown). A flow diagram of the process and the results are displayed in Figure 1.

A total of 460 biomarkers of aging were suggested in Round 1, which were reduced to 341 when categorized into major themes (Supplementary Table 2). Of these, 20 biomarkers were mentioned ≥10 times. These biomarkers were appraised by the panel in Round 2, and included: *physiological* (insulin-like growth factor 1 [IGF-1], growth-differentiating factor-15 [GDF-15], glucose, glycated hemoglobin [HbA1c], cholesterol), *inflammatory* (high sensitivity C-reactive protein [hsCRP], interleukin-6 [IL-6]), *functional* (muscle mass, muscle strength, hand grip strength [HGS], Timed-Up-and-Go [TUG], gait speed, standing balance test [SBT], frailty index, cognitive health, blood pressure), and *genetic*/*epigenetic* (telomere length, DNA methylation, epigenetic clocks) domains.

**Figure 2.**
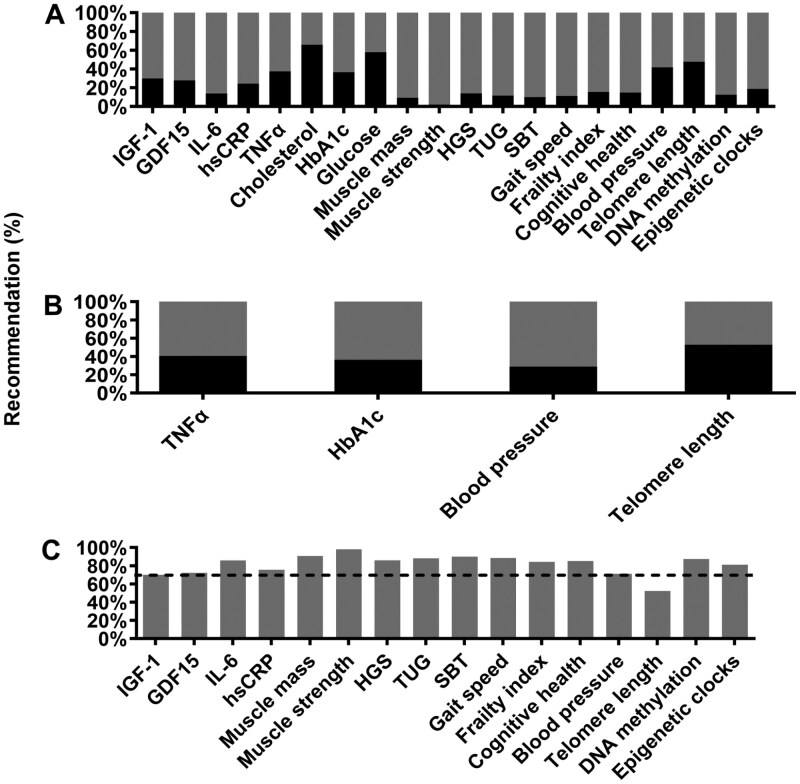
Summary of overall responses (yes/no) to biomarkers. (**A**) 20 biomarkers from Round 2, (**B**) 4 biomarkers recirculated for Round 3, and (**C**) Total recommended biomarkers. Black bars indicate % of responses denoted to not recommend the biomarker and dark gray bars indicate % of responses denoted to recommend the biomarker. Dashed line indicates the 70% threshold. GDF-15: growth differentiation factor 15; HbA1c: glycated hemoglobin; HGS: hand grip strength; hsCRP: high sensitivity C-reactive protein; IGF-1: insulin-like growth factor 1; IL-6: interleukin-6; SBT: standing balance test; TNF-α: tumor necrosis factor alpha; TUG: Timed-Up-and-Go.

In Round 2, 14 of the 20 potential biomarkers reached consensus. Two biomarkers were removed from further voting due to a lack of agreement, and 4 biomarkers were carried over to Round 3 for reevaluation (Figure 2A). Biomarkers that met consensus as a suitable biomarker of aging were: IGF-1, GDF-15, hsCRP, IL-6, muscle mass, muscle strength, HGS, TUG, gait speed, SBT, frailty index, cognitive health, and DNA methylation and epigenetic clocks which were merged into one biomarker for Round 3 due to similarity since epigenetic clocks typically use DNA methylation data. Biomarkers that attained less than 50% agreement between panel members, and thus were removed from further consideration, were cholesterol and glucose. Biomarkers reaching moderate consensus and further evaluated in Round 3 were TNF-α, HbA1c, blood pressure, and telomere length.

In Round 3, 1 biomarker (blood pressure) reached consensus and 3 biomarkers (TNF-α, HbA1c, telomere length) attained less than 70% agreement across 60 panel members (Figure 2B and C). Biomarkers which were not recommended (≤50% agreement) were glucose and cholesterol; biomarkers which had moderate agreement (51%–69% agreement) were TNF-α, HbA1c, and telomere length; and biomarkers that were recommended (≥70% agreement) were IGF-1, GDF-15, hsCRP, IL-6, muscle mass, muscle strength, HGS, TUG, gait speed, SBT, frailty index (eg, Fried, Rockwood Mitnitski), cognitive health (eg, Montreal cognitive assessment), blood pressure, and DNA methylation/epigenetic clocks. Full details of the level of agreement for the statements are listed in Supplementary Material (Supplementary Tables 4–23).

### Statements Achieving Consensus

**Figure 3.**
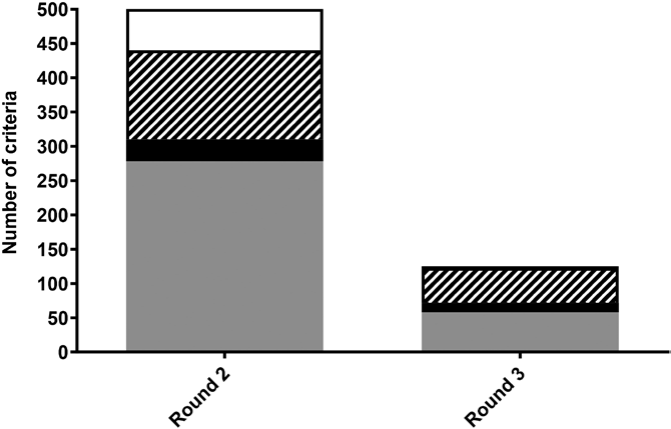
A summary of overall responses across Round 2 and Round 3. Statements are divided by those that were removed as agreement was ≤50% (white), those that were undecided as agreement was 51%–69% (striped), those that reached consensus for “No” with agreement at ≥70% (black), and those that reached consensus for “Yes” with agreement at ≥70% (dark gray).

Panel members were presented with a total of 500 statements for appraisal at the start of Round 2. In this round, 310 statements (62%) met consensus (90% “yes,” 10% “no”), 130 (26%) did not pass the threshold for consensus (51%–69% agreement), and 60 (12%) were removed due to either poor agreement between panel members (≤50% agreement) or because their associated biomarkers were removed from voting (Figure 3). As noted, prior to Round 3, two biomarkers “DNA methylation” and “epigenetic clocks” were amalgamated. When accounting for the amalgamation of these biomarkers (and thus the collapse of 50 statements associated with these biomarkers into 25 statements), the resulting total number of statements which met consensus, did not pass the threshold for consensus, and were removed was 291 (61%, 88% “yes,” 12% “no”), 125 (26%), and 59 (12%), respectively. Of the 125 statements re-appraised in Round 3, 72 (58%) met consensus (78% “yes,” 22% “no”), 51 (41%) did not pass the threshold for consensus, and 2 (1%) were removed. By Round 3, there was limited agreement (“yes”) on which biomarkers were suitable for use in acute (47%) and short-term (59%) interventions, while there was greater agreement on biomarkers suitable for use in medium (100%) and long-term (100%) interventions.

There was good agreement on the ease of use for different biomarkers (statements 5–7, 15 in Table 1), with 78%–100% of panel members providing the same response. For statements associated with biomarker mechanisms (statements 8, 21, 22) the agreement in responses ranged from 72% to 100%. Statements regarding the evaluation of the biomarker (statements 9–13, 15, 16 in Table 1) varied with complete agreement at the end of Round 3 for mechanistic validation, precision, reliability, and sensitivity (100%) to lower agreement for specificity (35%). Agreement on the ability to blind participants, researchers, and data analysts using the biomarkers (statements 18–20) ranged from 100% agreement for blinding to data analysts to 61% and 67% for blinding to researchers and participants, respectively.

**Figure 4.**
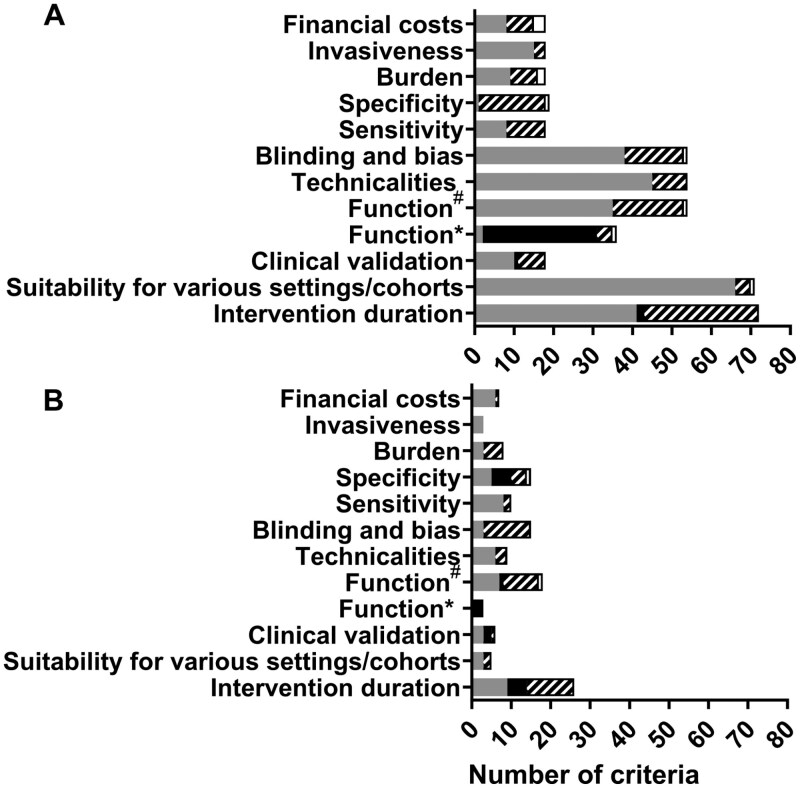
A summary of responses in each major theme in (**A**) Round 2 and (**B**) Round 3. Statements are divided by those that were removed as agreement was ≤50% (white), those that were undecided as agreement was 51%–69% (striped), those that reached consensus for “No” with agreement at ≥70% (black), and those that reached consensus for “Yes” with agreement at ≥70% (dark gray). Technicalities included precision, reliability, and mechanical validation; Function# included generalisabilty, prediction of biological age and responsiveness; Function* included requirement of models/software for, and age acceleration; Suitability for various settings included field, frail and cognitively impaired participants and access to materials; Intervention duration included acute, short, medium and long.

Most biomarkers were deemed to be either noninvasive (50%) or moderately invasive (50%), although there was less agreement (65%) on burden. Finally, agreement regarding financial cost varied depending upon the biomarker, although panel members typically agreed that there was minimal financial cost associated with physical function and blood-based biomarkers and higher costs associated with DNA methylation (Figure 4A and B). Based on the panel members’ recommendations a simple tool has been developed that can be used to select biomarkers based on suitability for use in different interventions/settings and can be found in the Supplementary Material (as an Excel file).

## Discussion

This study aimed to establish consensus on biomarkers of aging for use in human intervention studies. In total, 60 expert panel members completed all 3 iterative rounds. Consensus was reached for 14 biomarkers (88%) and 363 (76%) statements. Most of the biomarkers that achieved consensus were functional/physiological biomarkers, while consensus was also achieved for a limited number of molecular biomarkers (biological clock-based and inflammatory molecules).

### Statements Associated with Biomarker Recommendations

#### Intervention duration

All recommended biomarkers were deemed to be more suitable for medium- (3–6 months) and long-term (≥6 months) interventions than acute and short-term (< 3 months) interventions. The effects of any nutritional, lifestyle, or pharmacological intervention(s) may take time to occur and most current biomarkers of aging may be unable to detect changes in response to acute intervention studies. Furthermore, it is challenging to be certain that short-term changes in any outcome measure in response to an intervention provides information about the aging process, as many (if not most) of such changes are likely to be homeostatic responses with limited long-term significance.

#### Setting

All recommended biomarkers reached consensus on their suitability for frail and cognitively impaired participants. This is a crucial requirement if studies are to be carried out in aging populations, because frailty and cognitive impairment affect 12%–24% and 12%–41% of older adults (≥70 years) worldwide, respectively (31,32). Furthermore, most biomarkers were deemed suitable for field settings, apart from IL-6 and TNF-α, where the threshold for consensus was not met. This was unexpected, given some other blood-based biomarkers were deemed to be suitable for field testing and advancements in novel collection methods, such as dry blood spot sampling, means that IL-6 and TNF-α can be measured from samples collected in remote settings (33). However, one possible contributing factor toward the lack of consensus is that reliable detection of baseline concentrations for these markers requires ultra-sensitive methods that may be unavailable to some researchers compared with more standard laboratory techniques. The panel agreed that sampling and source materials could be easily obtained without the requirement for complex models for all biomarkers apart from DNA methylation/biological clock-based biomarkers (which were amalgamated for consideration in Round 3, given perceived overlap between these biomarkers); an expected finding since these molecular biomarkers require more advanced laboratory processes. There was, however, uncertainty regarding the use of complex models and software to assess cognitive health. This is potentially because cognitive health can be assessed using a variety of biomarkers ranging from paper-based and/or questionnaire-based cognitive tasks to state-of-the-art imaging and spectroscopy.

#### Functional link to aging

Collection or measurement of all the recommended biomarkers was not expected to influence the rate of aging. Most of the biomarkers were deemed generalizable across tissues and populations (3). Four biomarkers (hsCRP, TNF-α, HbA1c, and blood pressure) did not meet the agreement threshold for predicting biological age better than chronological age (3). Likewise, two biomarkers (HbA1c and blood pressure) did not meet the agreement threshold for being responsive biomarkers (ie, respond to accelerated or decelerated aging).

#### Assessment and technicalities

There was less agreement on the recommended biomarkers with regards to clinical validation, sensitivity, and specificity compared with mechanistic validation (ie, whether the biomarker reflects the underlying cellular and molecular mechanisms of aging). However, all biomarkers were deemed precise and reliable, with lower agreement for telomere length, perhaps because telomere length changes can be transient and may not reflect aging per se (34). Furthermore, there is large interindividual differences in telomere length, and multiple measurements over the lifetime of a participant may be required to make meaningful inferences about the aging trajectory (accepting that this would be associated with additional financial cost/participant burden and would require careful consideration of biological vs analytical variation when interpreting values).

#### Burden, invasiveness, and financial costs

Respondents considered that assessment of most biomarkers was associated with minimal burden, although 5 biomarkers (IL-6, TNF-α, muscle mass, cognitive health, DNA methylation) did not meet the agreement threshold. It is unclear why there was less agreement for IL-6 and TNF-α, while hsCRP, IGF-1, and GDF-15 were classified as minimal burden, despite it being possible to measure these biomarkers in plasma/serum. No biomarkers were suggested to be invasive; approximately half were deemed noninvasive and half moderately invasive. There was consensus that DNA methylation was associated with high financial cost while there was also consensus that the physiological biomarkers were low burden, noninvasive and of low financial cost; an expected finding given the minimal equipment required for assessment (35). Similarly, inflammatory blood-based biomarkers were perceived to have lower burden, lower invasiveness, and low financial cost, which mirrors recent consortia recommendations (3,6).

### Comparison with Other Studies and Reports

Of the 14 recommended biomarkers, muscle strength had the highest agreement (98%), while IGF-1 had the lowest (70%). The high number of physiological biomarker recommendations may have reflected the panel members’ expertise. Alternatively, it may be that these biomarkers are more appropriate and suitable for a range of statements and intervention settings than cellular/molecular biomarkers (35), or that the lack of consensus on other suggested biomarkers may be related to the inherent difficulties in evaluating their validity as aging biomarkers (17). Furthermore, research on cellular/molecular markers of aging is a rapidly evolving area with minimal time for each scientific development to mature and be useful to further clinical and research use. In contrast, physiological measures have been used in research and clinical contexts for many decades, and so may be more familiar to a broader range of individuals.

Previous groups and consortia have identified various biomarkers of aging. The MARK-AGE consortium and the Biomarker of Aging consortium have both recommended omics-based measures (epigenomics, transcriptomics, proteomics, and metabolomics) as potential biomarkers of aging (3,36). There were, however, fewer recommendations from our panel members in these categories. This may be because omics-based measures can be limited to advanced laboratory facilities and researchers with financial resources to support these approaches or because many of these promising cutting-edge biomarkers are still under investigation and there is a lack of specificity on exactly what should be assessed. Another category commonly recommended are inflammatory blood-based and hormone biomarkers such as IL-6, hsCRP, TNF-α (3,7,36–38), and GDF-15 (39), which were also suggested by our panel members. The Biomarkers of Ageing consortium among others (9–11,35,36,40) have proposed that physiological biomarkers could be suitable for measuring aging. Previous work (9,10,40) has encompassed physiological, metabolic (ie, HbA1c), physical capability, cognitive function, and social and psychological wellbeing in addition to utilizing the National Institutes of Health (NIH) toolbox (41) (an application consisting of over 100 validated tests allowing researchers to reliably assess cognitive, neuromuscular, sensory and emotional function throughout life). These recommendations were mirrored among our panel members with a high level of agreement for physiological biomarkers.

As highlighted in the FDA-NIH *Biomarkers, EndpointS, and other Tools (BEST)* (2), broad consensus has not yet been reached on the definitions of biomarker classes (ie, composite, digital) and their applications. Recent work by the Biomarkers of Ageing consortium is aiming to reach a consensus definition on these issues (3). Finally, as noted by others (2,3,7,36,42), the panel agreed that composite biomarkers are preferred over single biomarkers. This is in part due to the complexity and heterogeneity of the aging process across the body and between individuals (2,3,7,36,42) (discussed further below).

### Strengths and Limitations

The current study has several strengths. Firstly, we used an accepted scientific approach (the Delphi method) to pool the knowledge and expert opinions of panel members. Secondly, we recruited a large (greater in size compared with other multidisciplinary Delphi studies) (43,44), multi-national cohort of panel members with diverse expertise due to the broad inclusion criteria which captured a range of potentially different views. This diversity across the panel members was maintained throughout the study with no differences in those who completed partial rounds, or all 3 rounds. Thirdly, the study took an agnostic approach, in which specific biomarkers and their applications were proposed by panel members, rather than being suggested by the research team, to minimize researcher influence/bias. Fourthly, anonymity of the panel members was maintained across all rounds, which allowed individuals to express and change their responses privately without peer pressure (25,45,46). Such anonymity would not be possible if consensus were derived using other methods, such as via round table discussion. Fifthly, the online nature of the survey allowed panel members time to synthesize and process their thoughts and recommendations and allowed for a wide range of countries to be involved without restrictions imposed by travel and time-zone differences. Nevertheless, it is accepted that remotely conducting the study—as per the original Delphi method (18)—may have hindered communication for some individuals and increased the number of statements for which consensus was not reached, due to the inability to further clarify or discuss nuances in recommendations.

Several limitations should also be highlighted. Firstly, the study was conducted in English, and so non-English-speaking experts may have been unable to take part. This could have skewed the results toward practices or views more widely accepted among those with English language skills. However, during pilot testing, the readability and comprehensibility was assessed by a mix of non-native English and English-speaking researchers. Despite this limitation, this study had good international representation with panel members from 25 countries in Round 1 and 19 in Round 3 (of which 12% were from upper-middle income countries, and 4% from low-income countries (47)) and no evidence of selection/attrition bias between rounds. A second limitation is that, despite best efforts, our panel may not have been fully representative of the wider aging research community. The study had a larger proportion of panel members focusing on human physiology who were based in Europe (with a large proportion in the United Kngdom), and relatively poor representation from cellular biology, imaging, and clinical trials, which may have influenced the recommendations. Indeed, many of the biomarkers recommended in this study could be broadly classified as “physical and physiological function” biomarkers of aging. Such biomarkers may provide limited information about the underpinning molecular mechanisms of aging (48), which have been identified as important criteria for appropriate biomarkers of aging by some researchers (9–11,35,36,40). A third potential limitation is that we adopted one specific set of definitions for “aging” and “biomarkers of aging,” albeit ones that have been widely used in previous literature in this area. It is possible that using alternative definitions for “aging” and “biomarkers of aging” may have altered the final list of biomarkers of aging proposed in this study, although this possibility is minimized by the fact that the panel members were experts in the aging field.

### Recommendations and Future Research

To date, there is no clear consensus on a single biomarker to capture biological aging. Due to the complexity and nuances within biomarkers of aging research, as well as the heterogeneous nature of the aging process, with different organs/systems aging at different rates, it is unlikely that a single biomarker would capture the complex heterogeneous processes of aging. Thus, composite biomarkers, encompassing a range of biomarkers, may be the best way forward. While there is no existing consensus on the best combination of biomarkers to fully capture biological aging (3,7,10,36,49,50) addressing this research gap should be considered a priority for the future. Other promising approaches include the use of artificial intelligence and multimodal foundation models for data-driven outcomes and processing high-throughput omics methods (3). Our findings also reiterated the need to consider biomarkers that are suitable for field settings, such that they can be measured in resource-poor environments. Biobanks where biological specimens (and associated metadata) are stored for future testing are becoming more widespread and could offer an avenue for biomarker validation (3,9). However, this is likely to be associated with higher financial costs as some biomarkers (eg, omics-based) are less affordable, especially in studies with large sample sizes. There is also a need for standardized collection procedures and protocols for each recommended biomarker as seen in the NIH toolbox (41) to improve consistency between repeated measures and across populations (39,40). Repeated biomarker measurements in the same individual could provide information on the “pace of aging” (4) and the cross-validation across different populations could further help address the current gaps in biomarker validation (42). Finally, there is a need to identify or modify biomarkers of aging which can be measured earlier in life (eg, TUG may need to be adapted to prevent ceiling limits in younger, fitter adults) (10). This could help identify and address any potential risks which can be attenuated through lifestyle, nutritional, or pharmacological interventions (9,10).

## Conclusion

This study provides an international consensus on biomarkers of aging for use in human intervention studies. There was moderate to high consensus (70%–98% agreement) on 14 biomarkers (IGF-1, growth GDF-15, hsCRP, IL-6, muscle mass, muscle strength, hand grip strength, Timed-Up-and-Go, gait speed, standing balance test, frailty index, cognitive health, blood pressure, DNA methylation/epigenetic clocks) among panel members from a range of disciplines and countries. These findings may help harmonize outcome measures to facilitate the comparison of the intervention effectiveness across studies and aid in planning future interventions. Finally, the biomarkers recommended by the panel members may help shape future biomarker of aging guidelines and provide objective criteria for researchers in selecting the most appropriate, and economically viable biomarkers for a specific study.

## Supplementary Material

glae297_suppl_Supplementary_Data

## Data Availability

The data can be made available upon request and any further inquiries can be directed to the corresponding author, Giorgia.Perri@newcastle.ac.uk.
