# Supplementary material for: An Expert Consensus Statement on Biomarkers of Aging for Use in Intervention Studies
Source: J Gerontol A Biol Sci Med Sci. 2024 Dec 21;80(5):glae297. doi: 10.1093/gerona/glae297 (PMC11979094; doi:10.1093/gerona/glae297)
Supplement: glae297_suppl_Supplementary_Data [file glae297_suppl_supplementary_data.zip › Supplementary Material_071224.pdf]

Supplementary Material

eTable 1: List of societies and networks contacted via email or website contact forms during recruitment.
eTable 2: Demographics of the panel members (expertise, clinician, career stage, career location) across the three rounds.
eFigure 1: Pictorial representation of countries participating in Round 1 and Round 3.

eTable 3: A list of all biomarkers suggested by panel members during Round 1 of the Delphi method.

eFigure 2: Flow diagram of the Delphi process and results with indications of biomarkers and statements reaching consensus across each round. Numbers in parentheses indicate the numbers of statements reaching consensus (yes or no). *In Round 2, two biomarkers were amalgamated thus reducing the total number of statements from 500 to 475 and resulting in a total of 13 accepted (yes) by the end of Round 2, 125 undecided, and 59 removed biomarkers.

eTable 4: Responses to statements for insulin-like growth factor (IGF-1) across Round 2 and 3.

eTable 5: Responses to statements for growth differentiating factor 15 (GDF15) across Round 2 and 3.

eTable 6: Responses to statements for interleukin 6 (IL-6) across Round 2 and 3.

eTable 7: Responses to statements for high sensitivity C-reactive protein (hsCRP) across Round 2 and 3.

eTable 8: Responses to statements for tumour necrosis factor alpha (TNF-α) across Round 2 and 3.

eTable 9: Responses to statements for cholesterol across Round 2.

eTable 10: Responses to statements for glycated haemoglobin (HbA1c) across Round 2 and 3.

eTable 11: Responses to statements for glucose across Round 2.

eTable 12: Responses to statements for muscle mass across Round 2 and 3.

eTable 13: Responses to statements for muscle strength across Round 2 and 3.

eTable 14: Responses to statements for hand grip strength (HGS) across Round 2 and 3.

eTable 15: Responses to statements for Timed-Up-and-Go (TUG) across Round 2 and 3.

eTable 16: Responses to statements for standing balance test (SBT) across Round 2 and 3.

eTable 17: Responses to statements for gait speed across Round 2 and 3.

eTable 18: Responses to statements for frailty index across Round 2 and 3.

eTable 19: Responses to statements for cognitive health across Round 2 and 3.

eTable 20: Responses to statements for blood pressure across Round 2 and 3.

eTable 21: Responses to statements for telomere length across Round 2 and 3.

eTable 22: Responses to statements for DNA methylation across Round 2 and 3 (merged with epigenetic clocks).

eTable 23: Responses to statements for epigenetic clocks across Round 2 and 3 (merged with DNA methylation).

eTable 1: List of societies and networks contacted via email or website contact forms during recruitment.

American Federation Ageing Research	Federation of African Nutrition Societies (FANUS)	Network for Aging Research
Asia Pacific Clinical Nutrition Society (APCNS)	Federation of Asian Nutrition Societies (FANS)	Pandaomics
Biochemical Society	Future Blood Testing Network	Physiological Society
Biomega	GERAS Centre for Aging Research	Quebec Network for Research on Aging
British Society for Research on Ageing (BSRA)	Gerontology Research Centre	Research Centers Collab Network (NIA)
Buck Institute	Healthy Aging Program	Research Institute for Aging
Canadian Centre for Activity and Ageing	Hevolution	Researchers of English Longitudinal Study of Ageing (ELSA)
Canadian Institutes of Health Research Institute of Ageing	Inclinico	Royal Society of Biology
Centre de médecine préventive et d'activité physique	Insilico Medicine	Sociedad Latinoamericana de Nutrición (SLAN)
Centre for Ageing Research (C4AR)	Institute for Life Course and Aging	Society for Endocrinology
Centre for Aging and Brain Health Innovation	Institute on Aging and Lifelong Health	STAR Institute
Centre for Aging SMART	International Association of Gerontology and Geriatrics	The Micronutrient Forum
Centre for Elder Research	International Confederation of Dietetic Associations (ICDA)	The Royal College of Ophthalmologists
Centre for Osteoarthritis Pathogenesis	International Network of Food Data Systems (INFOODS)	Towards Understanding Longitudinal Studies in Older People (TULIPs)
Centre for Research in Aging	International Psychogeriatric Association	Trent Centre for Aging and Society
Centre for Studies in Ageing	Italian Physiological Society	UCLAN Global Health Institute
Centre of Excellence on Longevity	Leiden 85 Researchers	University of Pittsburgh Ageing Institute
Centre on Aging	LIFE Research Institute	Versus Arthritis
Centre on Aging and Health	McGill University Research Centre for Studies in Aging (MCSA)	World Obesity Federation
Chemistry42	McMaster Institute of Ageing Research	World Public Health Nutrition Association (WPHNA)
EuroAgeNet	Mexican Federation of Societies of Nutrition	Yale Public Health
	National Institute on Ageing (NIA)	York University Centre for Aging Research and Education (YUCARE)

eTable 2: Demographics of the panel members (expertise, clinician, career stage, career location) across the three rounds.

Variable		Round 1 Number (%)	Round 2 Number (%)	Round 3 Number (%)
**N**		116	87	60
**Expertise**	Ageing	44 (37.9)	34 (39.1)	20 (33.3)
	Physiology	17 (14.7)	10 (11.5)	7 (11.7)
	Nutrition	11 (9.5)	9 (10.3)	8 (13.3)
	Biomarkers	7 (6.0)	4 (4.6)	4 (6.7)
	Geriatric medicine	6 (5.2)	5 (5.7)	2 (3.3)
	Gerontology	5 (4.3)	5 (5.7)	4 (6.7)
	Endocrinology	2 (1.7)	2 (2.3)	2 (3.3)
	Epidemiology	2 (1.7)	1 (1.1)	1 (1.7)
	Geriatrics	2 (1.7)	1 (1.1)	1 (1.7)
	Geroscience	2 (1.7)	1 (1.1)	1 (1.7)
	Anti-Aging Genes	1 (0.9)	1 (1.1)	1 (1.7)
	Biochemistry	1 (0.9)	1 (1.1)	1 (1.7)
	Cell Biology	1 (0.9)		
	Clinical trials	1 (0.9)		
	Dementia	1 (0.9)	1 (1.1)	1 (1.7)
	Frailty	1 (0.9)	1 (1.1)	1 (1.7)
	General Practice	1 (0.9)	1 (1.1)	1 (1.7)
	Geriatric physiotherapist	1 (0.9)	1 (1.1)	1 (1.7)
	Histology	1 (0.9)	1 (1.1)	1 (1.7)
	Imaging	1 (0.9)	1 (1.1)	
	Metabolism	1 (0.9)	1 (1.1)	1 (1.7)
	Neuroscience	1 (0.9)	1 (1.1)	
	Nutrient sensing	1 (0.9)	1 (1.1)	1 (1.7)
	Omics	1 (0.9)		
	Physical Activity	1 (0.9)	1 (1.1)	1 (1.7)
	Primary care	1 (0.9)	1 (1.1)	
	Rehabilitation	1 (0.9)	1 (1.1)	
	Rheumatology	1 (0.9)	1 (1.1)	
**Age**		46.7±11.1 (n=93)	45.9±10.8 (n=70)	47.0±11.0 (n=49)
**Clinician**	Yes	33 (28.4)	26 (29.9)	16 (26.7)
	No	83 (71.6)	61 (70.1)	44 (73.3)
**Self-reported Career Stage**	Early	30 (25.9)	24 (27.6)	16 (26.7)
	Mid	34 (29.3)	28 (32.2)	19 (31.7)
	Senior	52 (44.8)	35 (40.2)	25 (41.7)
**Location of Work**	**Europe**	**77 (66.4)**	**59 (67.8)**	**39 (65.0)**
	UK	*55 (47.5)*	*43 (49.4)*	*26 (43.3)*
	Germany	*4 (3.4)*	*3 (3.4)*	*3 (5.0)*
	Italy	*3 (2.6)*	*1 (1.1)*	
	Spain	*3 (2.6)*	*3 (3.4)*	*3 (5.0)*
	Portugal	*2 (1.7)*	*2 (2.3)*	*2 (3.3)*
	Switzerland	*2 (1.7)*	*1 (1.1)*	*1 (1.7)*
	Austria	*1 (0.9)*	*1 (1.1)*	*1 (1.7)*
	Denmark	*1 (0.9)*		
	Finland	*1 (0.9)*	*1 (1.1)*	*1 (1.7)*
	France	*1 (0.9)*	*1 (1.1)*	*1 (1.7)*
	Greece	*1 (0.9)*	*1 (1.1)*	*1 (1.7)*
	Lithuania	*1 (0.9)*	*1 (1.1)*	
	Netherlands	*1 (0.9)*	*1 (1.1)*	
	Poland	*1 (0.9)*		
	USA	17 (14.7)	8 (9.2)	6 (10.0)

	Australia	4 (3.4)	4 (4.6)	3 (5.0)
	Canada	4 (3.4)	4 (4.6)	2 (3.3)
	China	3 (2.6)	3 (3.4)	1 (1.7)
	Indonesia	3 (2.6)	2 (2.3)	2 (3.3)
	New Zealand	2 (1.7)	2 (2.3)	2 (3.3)
	Singapore	2 (1.7)	2 (2.3)	2 (3.3)
	India	1 (0.9)	1 (1.1)	1 (1.7)
	Iran	1 (0.9)		
	Japan	1 (0.9)	1 (1.1)	1 (1.7)
	Russia	1 (0.9)	1 (1.1)	1 (1.7)

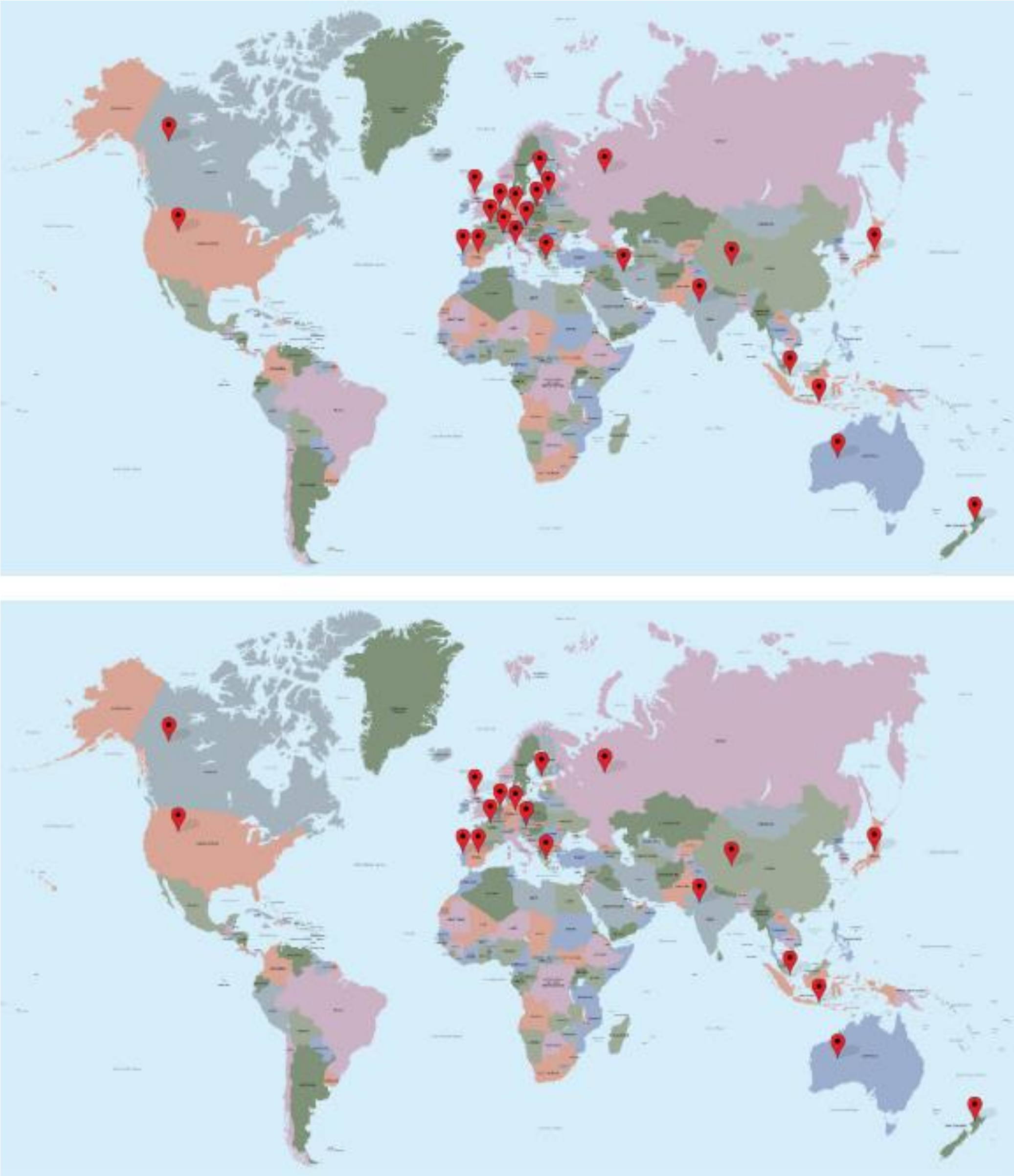

eFigure 1: Pictorial representation of countries participating in Round 1 and Round 3.

eTable 3: A list of all biomarkers suggested by panel members during Round 1 of the Delphi method.

Theme	Biomarkers	
**Clocks**	GrimAge	MetaboAge Score
	Dunedinpace/allostatic load	CausAge
	RetinalAge	DamAge
	BrainAge	AdaptAge
	PhenoAge	DNAmRS
	MetaboHealth Score	bAge
	Immune Age/iAge (CXCL9, TRAIL, IFNgamma, eotaxin, CXCL1)	SenMayo analysis
	Klemera-Doubal Biological Age	
**Epigenetic Markers**	DNA methylation (translated to downstream epigenetic clocks) > 3-month duration	Heterochromatin
	CpG methylation (gene ELOVL2, FHL2)	Disorganized 3D genome architecture
	Genome wide SNP (control for genetic predispositions)	Deregulated RNA modifications
	RNAseq in monocytes and natural killer cells	Genomic instability
	Histone methylation	Cell cycle arrest
	DNA methylation-based clocks (e.g. Horvath, Hannum)	microRNAs (small noncoding RNA)
	Epigenetic changes	H3K9me3, H3K27me3
**Imaging**	Brain imaging	Cross-sectional imaging for muscle
	Positron Emission Tomography (PET), 18FDG-PET	Cranial MRI for neurological age (front and parietal atrophy – brain age; temporal lobe and hippocampal atrophy), diffusion tensor imaging sequences (white matter hyperintensities)
	Imaging tracers related to ageing	FLAIR sequences
	3D facial imaging	Ultrasound (quadriceps thickness)
**Wearables**	Continuous glucose monitoring (CGM)	Mobility
	Autonomic tests (electrocardiogram, blood pressure)	
**Quality of life**	Self-rated health	Resilience
	Perceived health	
**Muscle**	Muscle strength (e.g., leg press)	Sarcopenia
	Muscle protein turnover (response to exercise and nutrition)	Creatinine
	Appendicular muscle mass (DXA, dual energy x-ray absorptiometry)	Myostatin
	Muscle echointensity	Urinary D3 Creatinine dilution
	Muscle motor unit activation	Muscle composition (IMAT)
	Muscle fibre type II to type I ratio	Intramuscular adipose tissue
**Anabolism**	Mammalian target of rapamycin (mTOR) (phosphorylation)	s6 kinase
	Ribosomal protein S6 kinase beta-1 (P70s6k1)	
**Frailty**	Fried frailty phenotype	Frailty index (Rockwood and Mitnitski)
**Bone**	Bone mineral density	Type III Procollagen Peptide (PIIINP)
	C-terminal telopeptide of type I collagen (CTX-I, CTX-II)	Serum procollagen type I N-propeptide (PINP)
**Physical function**	Walking speed (habitual)	Chair rise
	Spontaneous physical activity (step count)	Hand-grip strength
	Activities of daily living (Barthel)	Short physical performance battery (SPPB)
	Instrumental activities of daily living (Lawton)	Jump-plate technography measures (i.e., pMax, vMax)
	Timed-Up-and-Go (TUG)	VO_2max
	Standing balance test	Vital capacity

Theme	Biomarkers	
**Body composition (adjusted for sex)**	Body fat	Body mass index
	Fat-free mass	Body fat %
	Anthropometrics	Waist circumference
**Nutritional Status**	Prudent diet score	Folate
	Appetite	Vitamin C
	Diet quality index	Plasma Ferritin (females)
	Mini nutritional assessment	Oligosaccharides NA2F, NGA2F in plasma
	Magnesium	Plasma alpha-tocopherol (females)
	Vitamin D	Plasma lycopene (males)
	Carotenoids	Tryptophan
**Cardiovascular function**	Cardiolipins	Framingham Risk Score
	Sphingomyelins	N-terminal-pro B-type natriuretic peptide (NTproBNP)
	Vascular stiffness	ST-2 (soluble interleukin 1 receptor-like 1)
	Coronary arterial calcification	hs-Troponin T
	Blood pressure	Heart rate variability
	Disordered calcium	
**Function**	Endothelial	Lung (forced expiratory volume)
	Lysosomal (lipofuscin)	Antibody response to vaccine
**Mitochondrial function**	High resolution respirometry	Mitochondrial complex activity
	Nicotinamide adenine dinucleotide (NAD^+)	Mitochondrial respiration (T-cells, monocytes)
	PGC1 alpha expression	
**Degeneration**	Spleen	Lymph nodes
	Thymus	
**Dysregulation**	Mahalonobis distance (homeostatic dysregulation)	Aberrant signalling pathways
	Allosteric load	Loss of proteostasis
	Nuclear body disorder	
Blood Biomarkers		
**Lipid**	Triglycerides	High density lipoprotein (HDL)
	Cholesterol	Apolipoprotein E
**Metabolic function**	Glycated haemoglobin (HbA1c)	Uric acid
	Insulin	Glycated serum proteins (GSP)
	Glucose	α-Ketoglutarate
	C-peptide	
	White blood cell count	Sirtuins ((silent mating type information regulation 2 homolog) 1)
	Lymphocytes	Serum alpha-2-macroglobulin (males)
	Albumin	Prostate specific antigen (males)
	Klotho	Lipoproteins
	Plasma Caveolin-1	
**Chem24**	Albumin, Alkaline Phosphatase, Alanine Amino Transferase, Aspartate Amino Transferase, Bicarbonate, Urea Nitrogen, Calcium, Chloride, Creatinine, Glucose, Potassium, Sodium, Total Bilirubin, Direct Bilirubin, Indirect Bilirubin, Gamma Glutamyl Transferase	Lactate Dehydrogenase, Total Protein, Anion Gap, Uric Acid, Phosphorus, Cholesterol, Triglycerides, LDL Cholesterol, albumin/globulin ratio, and blood urea nitrogen/Creatinine Ratio
**Endocrine**	Liver function (Alkaline phosphatase (ALP) Alanine aminotransferase (ALT)	Hormones – sex steroids (oestrogen, testosterone, sex hormone binding globulin), growth hormone, DHEAs, insulin-like growth factors (IGF-1), thyroid, Fibroblast growth factor 21 (FGF21), adiponectin, leptin
	Kidney eGFR, creatine kinase	Wechler Digit-Symbol Task

Theme	Biomarker	
**Cognitive function**	Executive memory	Neurofilament light chain (NfL)
	Global cognition	Trail Making Test (TMT)
	Episodic memory (cued recall and AVLT)	Plasma t-tau and p-tau
	Fine motor ability (spiral drawing and pegboard test)	Plasma CSF sTREM2
	Standardised mini mental state examination	Plasma GFAP
	Fluid intelligence measures	Plasma ALCAM, FSH or NAD+/NADH, GDF11 and GAPDH mRNA
	Cerebral blood flow	White matter integrity
	Memory/Naive CD4 ratio, Memory/Naive B cell ratio	Amyloid-β
	CD4/CD8 ratio	Glial fibrillary acidic protein (GFAP)
	Montreal Cognitive Assessment	Brain-derived neurotrophic factor (BDNF)
	Skin autofluorescence	CK19
**Stem cell**	Exhaustion - clonal haematopoiesis of indeterminate potential (CHIP)	CD34+
	Oct-4	
**Inflammation/immune**	Cytokines	Fas
	Growth factors (IGF1, IGF3, IGFBP, GDF15, IGFBP1)	Osteopontin
	Interleukins (IL-15, IL-6, IL-8, IL-5, IL-1, 1β)	CCL3
	Interferon-gamma inducible protein of 10 kDa (IP-10)	Fibrinogen
	Activin A	Immunosenescent cells
	PAI-1/serpins	Inflammaging composite indices
	Matrix metalloproteinases (MMP)	T cell subsets
	Advanced Glycation End Products (AGEs)	Neutrophil migratory dynamics
	Immune cell proportions	High mobility group box 1 (HMGB1)
**Antioxidant related**	Total antioxidant capacity (TAC)	Malondialdehyde (MDA)
	Reactive oxygen metabolites (ROM)	4-Hydroxynonenal (4 HNE)
	Superoxide Dismutase (SOD)	Protein carbonyls
	3-nitrotyrosine	
**Extracellular Matrix components**	Collagen, focal adhesion components	
**Senescence**	B-galactosidase	CDKN2A/p16
	SASP factors	Replicative senescence
**Chromatin remodelling**	Senescence-associated heterochromatin foci	
**Cellular**	Cell free DNA	FOXO3 expression
	gamma-H2AX	TNF-β
	Lamin B1	Wnt signalling
**Telomere**	Length (whole blood, monocytes, leukocytes, T-cells, hematopoietic stem cells, smooth tissues – liver, stomach, intestine)	Attrition
	Stability	
**Omics**	Proteomics	Lipidomics
	Methylomics	Microbiome
	Metabolomics (plasma and urine)	

CXCL9: Chemokine (C-X-C motif) ligand 9; CXCL1: The chemokine (C-X-C motif) ligand 1; FHL2: Four And A Half LIM Domains 2; ELOVL2: Elongation Of Very Long Chain Fatty Acids-Like 2; H3K9me3: Trimethylation of histone H3 lysine 9; FLAIR: Fluid attenuated inversion recovery; PGC1: Peroxisome proliferator-activated receptor-gamma coactivator; CD"X": cluster of differentiation X; CK19: cytokeratin 19; Oct-4: octamer-binding transcription factor 4; IGF-"X": insulin-like growth factor X; GDF15: growth differentiating factor 15; IL-"X": interleukin-X; PAI-1: Plasminogen activator inhibitor-1; CCL3: Chemokine (C-C motif) ligand 3; SASP: Senescence-associated secretory phenotype; TNF-B: tumour necrosis factor beta; CDKN2A: cyclin-dependent kinase inhibitor 2A; FOXO3: Forkhead box O; gamma-H2AX: gamma H2A histone family member X; TNF-β: Tumor necrosis factor-beta

eTable 4: Responses to statements for insulin-like growth factor (IGF-1) across Round 2 and 3.

Insulin-like growth factor (IGF-1)	Round 2			Round 3		
**Statements**	**n**	**Yes**	**No**	**n**	**Yes**	**No**
Suitable as an outcome for acute intervention studies?	29	37.9	62.1	23	30.4	69.6
Suitable as an outcome for short term intervention (< 3 months) studies?	29	48.3	51.7	23	43.5	56.5
Suitable as an outcome for medium term intervention (3-6 months) studies?	29	72.4	27.6			
Suitable as an outcome for long term intervention (≥ 6 months) studies?	29	75.9	24.1			
Suitable for field settings?	30	73.3	26.7			
Suitable for cognitively impaired participants?	30	86.7	13.3			
Suitable for frail participants?	30	93.3	6.7			
Does the act of measuring this biomarker accelerate ageing?	30	23.3	76.7			
Is it clinically validated (i.e., has it been validated for use in clinical settings against set clinical standards)?	29	62.1	37.9	22	77.3	22.7
Is it mechanistically validated (i.e., does the biomarker reflect underlying cellular and molecular mechanisms of ageing)?	29	86.2	13.8			
Is it generalisable (i.e., does the biomarker function across different applications i.e., cell type, organ, system, human populations)?	30	70.0	30.0			
Is it precise (i.e., repeatable and reproducible)?	30	86.7	13.3			
Is it reliable (i.e., repeatable with minimal technical variability)?	30	90.0	10.0			
Are sampling and source materials easily obtained including collection, storage and processing?	30	96.7	3.3			
Are complex models or software required for interpretation?	30	3.3	96.7			
Is it sensitive?	29	69.0	31.0	22	77.3	22.7
Is it specific?	29	48.3	51.7	22	50.0	50.0
Can it be blinded to participants?	30	100.0	0.0			
Can it be blinded to researchers?	30	100.0	0.0			
Can it be blinded to data analysts?	29	93.1	6.9			
Does it predict functional aspects of ageing better than chronological ageing?	29	48.3	51.7	22	50.0	50.0
Is it responsive (i.e., does it respond to accelerated or decelerated ageing)?	28	75.0	25.0			
Is this biomarker...	30					
• Minimal burden		70.0				
• Moderate burden		30.0				
• Burdensome		0.0				
Is this biomarker...	30					
• Non-invasive		16.7				
• Moderately invasive		73.3				
• Invasive		10.0				
Is this biomarker...	29					
• Minimal financial cost (< $10/participant)		13.8				
• Low financial cost ($10-50/participant)		75.9				
• Moderate financial cost ($51-100/participant)		10.3				
• High financial cost ($101-1000+ /participant)		0.0				

eTable 5: Responses to statements for growth differentiating factor 15 (GDF15) across Round 2 and 3.

Growth differentiating factor 15 (GDF15)	Round 2			Round 3		
Statements	n	Yes	No	n	Yes	No
Suitable as an outcome for acute intervention studies?	16	37.5	62.5	12	16.7	83.3
Suitable as an outcome for short term intervention (< 3 months) studies?	15	46.7	53.3	12	33.3	66.7
Suitable as an outcome for medium term intervention (3-6 months) studies?	15	73.3	26.7			
Suitable as an outcome for long term intervention (≥ 6 months) studies?	15	80.0	20.0			
Suitable for field settings?	16	81.3	18.8			
Suitable for cognitively impaired participants?	17	88.2	11.8			
Suitable for frail participants?	17	94.1	5.9			
Does the act of measuring this biomarker accelerate ageing?	17	35.3	64.7	14	21.4	78.6
Is it clinically validated (i.e., has it been validated for use in clinical settings against set clinical standards)?	16	37.3	62.5	13	23.1	76.9
Is it mechanistically validated (i.e., does the biomarker reflect underlying cellular and molecular mechanisms of ageing)?	16	81.3	18.8			
Is it generalisable (i.e., does the biomarker function across different applications i.e., cell type, organ, system, human populations)?	16	81.3	18.8			
Is it precise (i.e., repeatable and reproducible)?	16	87.5	12.5			
Is it reliable (i.e., repeatable with minimal technical variability)?	16	87.5	12.5			
Are sampling and source materials easily obtained including collection, storage, and processing?	16	100.0	0.0			
Are complex models or software required for interpretation?	15	13.3	86.7			
Is it sensitive?	16	87.5	12.5			
Is it specific?	16	50.0	50.0			
Can it be blinded to participants?	15	100.0	0.0			
Can it be blinded to researchers?	15	93.3	6.7			
Can it be blinded to data analysts?	15	93.3	6.7			
Does it predict functional aspects of ageing better than chronological ageing?	16	81.3	18.8			
Is it responsive (i.e., does it respond to accelerated or decelerated ageing)?	15	86.7	13.3			
Is this biomarker...	17					
• Minimal burden		76.5				
• Moderate burden		23.5				
• Burdensome		0.0				
Is this biomarker...	18					
• Non-invasive		27.8				
• Moderately invasive		72.2				
• Invasive		0.0				
Is this biomarker...	17					
• Minimal financial cost (< $10/participant)		5.9				
• Low financial cost ($10-50/participant)		76.5				
• Moderate financial cost ($51-100/participant)		11.8				
• High financial cost ($101-1000+ /participant)		5.9				

eTable 6: Responses to statements for interleukin 6 (IL-6) across Round 2 and 3.

Interleukin 6 (IL-6)	Round 2			Round 3		
Statements	n	Yes	No	n	Yes	No
Suitable as an outcome for acute intervention studies?	40	65.0	35.0	33	87.9	12.1
Suitable as an outcome for short term intervention (< 3 months) studies?	40	77.5	22.5			
Suitable as an outcome for medium term intervention (3-6 months) studies?	38	73.7	26.3			
Suitable as an outcome for long term intervention (≥ 6 months) studies?	38	73.7	26.3			
Suitable for field settings?	41	65.9	34.1	33	60.6	39.4
Suitable for cognitively impaired participants?	39	87.2	12.8			
Suitable for frail participants?	40	92.5	7.5			
Does the act of measuring this biomarker accelerate ageing?	41	19.5	80.5			
Is it clinically validated (i.e., has it been validated for use in clinical settings against set clinical standards)?	40	67.5	32.5	32	75.0	25.0
Is it mechanistically validated (i.e., does the biomarker reflect underlying cellular and molecular mechanisms of ageing)?	41	87.8	12.2			
Is it generalisable (i.e., does the biomarker function across different applications i.e., cell type, organ, system, human populations)?	41	80.5	19.5			
Is it precise (i.e., repeatable and reproducible)?	40	75.0	25.0			
Is it reliable (i.e., repeatable with minimal technical variability)?	40	87.5	12.5			
Are sampling and source materials easily obtained including collection, storage and processing?	41	95.1	4.9			
Are complex models or software required for interpretation?	41	9.8	90.2			
Is it sensitive?	40	72.5	27.5			
Is it specific?	41	31.7	68.3	33	21.2	78.8
Can it be blinded to participants?	40	97.5	2.5			
Can it be blinded to researchers?	39	97.4	2.6			
Can it be blinded to data analysts?	40	92.5	7.5			
Does it predict functional aspects of ageing better than chronological ageing?	40	62.5	37.5	33	21.2	78.8
Is it responsive (i.e., does it respond to accelerated or decelerated ageing)?	37	73.0	27.0			
Is this biomarker...	41			33		
• Minimal burden		63.4			66.7	
• Moderate burden		34.1			30.3	
• Burdensome		2.4			3.0	
Is this biomarker...	40			33		
• Non-invasive		20.0			15.2	
• Moderately invasive		67.5			81.8	
• Invasive		12.5			3.0	
Is this biomarker...	41			33		
• Minimal financial cost (< $10/participant)		26.8			15.2	
• Low financial cost ($10-50/participant)		56.1			75.8	
• Moderate financial cost ($51-100/participant)		17.1			9.1	
• High financial cost ($101-1000+ /participant)		0.0			0.0	

eTable 7: Responses to statements for high sensitivity C-reactive protein (hsCRP) across Round 2 and 3.

High sensitivity C-reactive protein (hsCRP)	Round 2			Round 3		
**Statements**	**n**	**Yes**	**No**	**n**	**Yes**	**No**
Suitable as an outcome for acute intervention studies?	39	64.1	35.9	30	86.7	13.3
Suitable as an outcome for short term intervention (< 3 months) studies?	40	75.0	25.0			
Suitable as an outcome for medium term intervention (3-6 months) studies?	38	73.7	26.3			
Suitable as an outcome for long term intervention (≥ 6 months) studies?	40	70.0	30.0			
Suitable for field settings?	41	78.0	22.0			
Suitable for cognitively impaired participants?	41	85.4	14.6			
Suitable for frail participants?	41	92.7	7.3			
Does the act of measuring this biomarker accelerate ageing?	41	17.1	82.9			
Is it clinically validated (i.e., has it been validated for use in clinical settings against set clinical standards)?	41	87.8	12.2			
Is it mechanistically validated (i.e., does the biomarker reflect underlying cellular and molecular mechanisms of ageing)?	41	70.7	29.3			
Is it generalisable (i.e., does the biomarker function across different applications i.e., cell type, organ, system, human populations)?	40	72.5	27.5			
Is it precise (i.e., repeatable and reproducible)?	41	87.8	12.2			
Is it reliable (i.e., repeatable with minimal technical variability)?	41	95.1	4.9			
Are sampling and source materials easily obtained including collection, storage, and processing?	41	95.1	4.9			
Are complex models or software required for interpretation?	41	9.8	90.2			
Is it sensitive?	41	80.5	19.5			
Is it specific?	41	31.7	68.3	31	9.7	90.3
Can it be blinded to participants?	40	100.0	0.0			
Can it be blinded to researchers?	40	100.0	0.0			
Can it be blinded to data analysts?	40	100.0	0.0			
Does it predict functional aspects of ageing better than chronological ageing?	40	57.5	42.5	29	58.6	41.4
Is it responsive (i.e., does it respond to accelerated or decelerated ageing)?	39	71.8	28.2			
Is this biomarker...	41			30		
• Minimal burden		63.4			80.0	
• Moderate burden		36.6			20.0	
• Burdensome		0.0			0.0	
Is this biomarker...	41			31		
• Non-invasive		19.5			12.9	
• Moderately invasive		68.3			83.9	
• Invasive		12.2			3.2	
Is this biomarker...	41			31		
• Minimal financial cost (< $10/participant)		61.0			71.0	
• Low financial cost ($10-50/participant)		39.0			29.0	
• Moderate financial cost ($51-100/participant)		0.0			0.0	
• High financial cost ($101-1000+ /participant)		0.0			0.0	

eTable 8: Responses to statements for tumour necrosis factor alpha (TNF-α) across Round 2 and 3.

Tumour necrosis factor alpha (TNF-α)	Round 2			Round 3		
**Statements**	**n**	**Yes**	**No**	**n**	**Yes**	**No**
**Suitable as an outcome for acute intervention studies?**	30	60.0	40.0	25	72.0	28.0
**Suitable as an outcome for short term intervention (< 3 months) studies?**	30	66.7	33.3	25	68.0	32.0
**Suitable as an outcome for medium term intervention (3-6 months) studies?**	29	65.5	34.5	25	76.0	24.0
**Suitable as an outcome for long term intervention (≥ 6 months) studies?**	29	62.1	37.9	25	72.0	28.0
**Suitable for field settings?**	30	66.7	33.3	25	56.0	44.0
**Suitable for cognitively impaired participants?**	31	87.1	12.9			
**Suitable for frail participants?**	31	90.3	9.7			
**Does the act of measuring this biomarker accelerate ageing?**	31	25.8	74.2			
**Is it clinically validated (i.e., has it been validated for use in clinical settings against set clinical standards)?**	30	56.7	43.3	24	62.5	37.5
**Is it mechanistically validated (i.e., does the biomarker reflect underlying cellular and molecular mechanisms of ageing)?**	30	70.0	30.0			
**Is it generalisable (i.e., does the biomarker function across different applications i.e., cell type, organ, system, human populations)?**	30	76.7	23.3			
**Is it precise (i.e., repeatable and reproducible)?**	31	80.6	19.4			
**Is it reliable (i.e., repeatable with minimal technical variability)?**	31	80.6	19.4			
**Are sampling and source materials easily obtained including collection, storage, and processing?**	31	87.1	12.9			
**Are complex models or software required for interpretation?**	31	9.7	90.3			
**Is it sensitive?**	30	70.0	30.0			
**Is it specific?**	29	31.0	69.0	25	12.0	88.0
**Can it be blinded to participants?**	30	100.0	0.0			
**Can it be blinded to researchers?**	29	100.0	0.0			
**Can it be blinded to data analysts?**	30	96.7	3.3			
**Does it predict functional aspects of ageing better than chronological ageing?**	31	51.6	48.4	26	53.8	46.2
**Is it responsive (i.e., does it respond to accelerated or decelerated ageing)?**	28	64.3	35.7	23	82.6	17.4
**Is this biomarker...**	32			26		
• **Minimal burden**		59.4			61.5	
• **Moderate burden**		37.5			38.5	
• **Burdensome**		3.1			0.0	
**Is this biomarker...**	32					
• **Non-invasive**		18.8				
• **Moderately invasive**		71.9				
• **Invasive**		9.4				
**Is this biomarker...**	31			25		
• **Minimal financial cost (< $10/participant)**		25.8			16.0	
• **Low financial cost ($10-50/participant)**		58.1			80.0	
• **Moderate financial cost ($51-100/participant)**		16.1			4.0	
• **High financial cost ($101-1000+ /participant)**		0.0			0.0	

eTable 9: Responses to statements for cholesterol across Round 2.

Cholesterol	Round 2		
**Statements**	**n**	**Yes**	**No**
**Suitable as an outcome for acute intervention studies?**	38	21.1	78.9
**Suitable as an outcome for short term intervention (< 3 months) studies?**	38	39.5	60.5
**Suitable as an outcome for medium term intervention (3-6 months) studies?**	38	57.9	42.1
**Suitable as an outcome for long term intervention (≥ 6 months) studies?**	38	63.2	36.8
**Suitable for field settings?**	39	76.9	23.1
**Suitable for cognitively impaired participants?**	39	87.2	12.8
**Suitable for frail participants?**	39	87.2	12.8
**Does the act of measuring this biomarker accelerate ageing?**	39	17.9	82.1
**Is it clinically validated (i.e., has it been validated for use in clinical settings against set clinical standards)?**	39	79.5	20.5
**Is it mechanistically validated (i.e., does the biomarker reflect underlying cellular and molecular mechanisms of ageing)?**	38	28.9	71.1
**Is it generalisable (i.e., does the biomarker function across different applications i.e., cell type, organ, system, human populations)?**	38	44.7	55.3
**Is it precise (i.e., repeatable and reproducible)?**	39	89.7	10.3
**Is it reliable (i.e., repeatable with minimal technical variability)?**	39	89.7	10.3
**Are sampling and source materials easily obtained including collection, storage, and processing?**	39	94.9	5.1
**Are complex models or software required for interpretation?**	39	2.6	97.4
**Is it sensitive?**	39	56.4	43.6
**Is it specific?**	39	48.7	51.3
**Can it be blinded to participants?**	37	94.6	5.4
**Can it be blinded to researchers?**	37	94.6	5.4
**Can it be blinded to data analysts?**	37	94.6	5.4
**Does it predict functional aspects of ageing better than chronological ageing?**	38	23.7	76.3
**Is it responsive (i.e., does it respond to accelerated or decelerated ageing)?**	39	33.3	66.7
**Is this biomarker...**	40		
• **Minimal burden**		82.5	
• **Moderate burden**		17.5	
• **Burdensome**		0.0	
**Is this biomarker...**	40		
• **Non-invasive**		25.0	
• **Moderately invasive**		65.0	
• **Invasive**		10.0	
**Is this biomarker...**	39		
• **Minimal financial cost (< $10/participant)**		69.2	
• **Low financial cost ($10-50/participant)**		28.2	
• **Moderate financial cost ($51-100/participant)**		2.6	
• **High financial cost ($101-1000+ /participant)**		0.0	

eTable 10: Responses to statements for glycated haemoglobin (HbA1c) across Round 2 and 3.

Glycated haemoglobin (HbA1c)	Round 2			Round 3		
Statements	n	Yes	No	n	Yes	No
Suitable as an outcome for acute intervention studies?	39	25.6	74.4			
Suitable as an outcome for short term intervention (< 3 months) studies?	39	48.7	51.3	33	51.5	48.5
Suitable as an outcome for medium term intervention (3-6 months) studies?	39	82.1	17.9			
Suitable as an outcome for long term intervention (≥ 6 months) studies?	39	84.6	15.4			
Suitable for field settings?	40	75.0	25.0			
Suitable for cognitively impaired participants?	40	92.5	7.5			
Suitable for frail participants?	40	95.0	5.0			
Does the act of measuring this biomarker accelerate ageing?	40	17.5	82.5			
Is it clinically validated (i.e., has it been validated for use in clinical settings against set clinical standards)?	40	90.0	10.0			
Is it mechanistically validated (i.e., does the biomarker reflect underlying cellular and molecular mechanisms of ageing)?	39	59.0	41.0	39	73.5	26.5
Is it generalisable (i.e., does the biomarker function across different applications, i.e., cell type, organ, system, human populations)?	40	57.5	42.5	40	70.6	29.4
Is it precise (i.e., repeatable and reproducible)?	40	95.0	5.0			
Is it reliable (i.e., repeatable with minimal technical variability)?	40	97.5	2.5			
Are sampling and source materials easily obtained including collection, storage, and processing?	38	97.4	2.6			
Are complex models or software required for interpretation?	40	0.0	100.0			
Is it sensitive?	40	82.5	17.5			
Is it specific?	40	62.5	37.5	34	70.6	29.4
Can it be blinded to participants?	39	97.4	2.6			
Can it be blinded to researchers?	39	97.4	2.6			
Can it be blinded to data analysts?	38	97.4	2.6			
Does it predict functional aspects of ageing better than chronological ageing?	38	44.7	55.3	32	34.4	65.6
Is it responsive (i.e., does it respond to accelerated or decelerated ageing)?	38	52.6	47.4	33	54.5	45.5
Is this biomarker...	40					
• Minimal burden		70.0				
• Moderate burden		27.5				
• Burdensome		2.5				
Is this biomarker...	40					
• Non-invasive		20.0				
• Moderately invasive		70.0				
• Invasive		10.0				
Is this biomarker...	39			34		
• Minimal financial cost (< $10/participant)		46.2			38.2	
• Low financial cost ($10-50/participant)		53.8			61.8	
• Moderate financial cost ($51-100/participant)		0.0			0.0	
• High financial cost ($101-1000+ /participant)		0.0			0.0	

eTable 11: Responses to statements for glucose across Round 2.

Glucose	Round 2		
**Statements**	**n**	**Yes**	**No**
**Suitable as an outcome for acute intervention studies?**	40	65.0	35.0
**Suitable as an outcome for short term intervention (< 3 months) studies?**	39	59.0	41.0
**Suitable as an outcome for medium term intervention (3-6 months) studies?**	40	57.5	42.5
**Suitable as an outcome for long term intervention (≥ 6 months) studies?**	40	65.0	35.0
**Suitable for field settings?**	40	80.0	20.0
**Suitable for cognitively impaired participants?**	39	84.6	15.4
**Suitable for frail participants?**	40	85.0	15.0
**Does the act of measuring this biomarker accelerate ageing?**	40	12.5	87.5
**Is it clinically validated (i.e., has it been validated for use in clinical settings against set clinical standards)?**	40	80.0	20.0
**Is it mechanistically validated (i.e., does the biomarker reflect underlying cellular and molecular mechanisms of ageing)?**	40	60.0	40.0
**Is it generalisable (i.e., does the biomarker function across different applications i.e., cell type, organ, system, human populations)?**	40	65.0	35.0
**Is it precise (i.e., repeatable and reproducible)?**	40	90.0	10.0
**Is it reliable (i.e., repeatable with minimal technical variability)?**	39	89.7	10.3
**Are sampling and source materials easily obtained including collection, storage, and processing?**	40	97.5	2.5
**Are complex models or software required for interpretation?**	40	0.0	100.0
**Is it sensitive?**	40	82.5	17.5
**Is it specific?**	40	47.5	52.5
**Can it be blinded to participants?**	38	94.7	5.3
**Can it be blinded to researchers?**	39	94.9	5.1
**Can it be blinded to data analysts?**	39	92.3	7.7
**Does it predict functional aspects of ageing better than chronological ageing?**	40	32.5	67.5
**Is it responsive (i.e., does it respond to accelerated or decelerated ageing)?**	40	40.0	60.0
**Is this biomarker...**	41		
• **Minimal burden**		75.6	
• **Moderate burden**		22.0	
• **Burdensome**		2.4	
**Is this biomarker...**	41		
• **Non-invasive**		29.3	
• **Moderately invasive**		58.5	
• **Invasive**		12.2	
**Is this biomarker...**	40		
• **Minimal financial cost (< $10/participant)**		77.5	
• **Low financial cost ($10-50/participant)**		22.5	
• **Moderate financial cost ($51-100/participant)**		0.0	
• **High financial cost ($101-1000+ /participant)**		0.0	

eTable 12: Responses to statements for muscle mass across Round 2 and 3.

Muscle strength	Round 2			Round 3		
**Statements**	**n**	**Yes**	**No**	**n**	**Yes**	**No**
**Suitable as an outcome for acute intervention studies?**	52	42.3	57.7	38	26.3	73.7
**Suitable as an outcome for short term intervention (< 3 months) studies?**	52	59.6	40.4	39	82.1	17.9
**Suitable as an outcome for medium term intervention (3-6 months) studies?**	51	86.3	13.7			
**Suitable as an outcome for long term intervention (≥ 6 months) studies?**	52	94.2	5.8			
**Suitable for field settings?**	52	75.0	25.0			
**Suitable for cognitively impaired participants?**	51	84.3	15.7			
**Suitable for frail participants?**	52	94.2	5.8			
**Does the act of measuring this biomarker accelerate ageing?**	52	13.5	86.5			
**Is it clinically validated (i.e., has it been validated for use in clinical settings against set clinical standards)?**	52	80.8	19.2			
**Is it mechanistically validated (i.e., does the biomarker reflect underlying cellular and molecular mechanisms of ageing)?**	52	86.5	13.5			
**Is it generalisable (i.e., does the biomarker function across different applications, i.e., cell type, organ, system, human populations)?**	52	59.6	40.4	39	69.2	30.8
**Is it precise (i.e., repeatable and reproducible)?**	52	90.4	9.6			
**Is it reliable (i.e., repeatable with minimal technical variability)?**	52	84.6	15.4			
**Are sampling and source materials easily obtained including collection, storage, and processing?**	50	66.0	34.0	38	73.7	26.3
**Are complex models or software required for interpretation?**	52	30.8	69.2	38	26.3	73.7
**Is it sensitive?**	51	76.5	23.5			
**Is it specific?**	51	62.7	37.3	37	70.3	29.7
**Can it be blinded to participants?**	51	72.5	27.5			
**Can it be blinded to researchers?**	50	56.0	44.0	36	61.1	38.9
**Can it be blinded to data analysts?**	50	90.0	10.0			
**Does it predict functional aspects of ageing better than chronological ageing?**	51	88.2	11.8			
**Is it responsive (i.e., does it respond to accelerated or decelerated ageing)?**	50	92.0	8.0			
**Is this biomarker...**	53			39		
• **Minimal burden**		50.9			66.7	
• **Moderate burden**		34.0			30.8	
• **Burdensome**		15.1			2.6	
**Is this biomarker...**	53					
• **Non-invasive**		81.1				
• **Moderately invasive**		17.0				
• **Invasive**		1.9				
**Is this biomarker...**	52					
• **Minimal financial cost (< $10/participant)**		34.6				
• **Low financial cost ($10-50/participant)**		23.1				
• **Moderate financial cost ($51-100/participant)**		23.1				
• **High financial cost ($101-1000+ /participant)**		19.2				

eTable 13: Responses to statements for muscle strength across Round 2 and 3.

Muscle strength	Round 2			Round 3		
**Statements**	**n**	**Yes**	**No**	**n**	**Yes**	**No**
**Suitable as an outcome for acute intervention studies?**	50	62.0	38.0	36	75.0	25.0
**Suitable as an outcome for short term intervention (< 3 months) studies?**	50	76.0	24.0			
**Suitable as an outcome for medium term intervention (3-6 months) studies?**	50	94.0	6.0			
**Suitable as an outcome for long term intervention (≥ 6 months) studies?**	50	98.0	2.0			
**Suitable for field settings?**	50	94.0	6.0			
**Suitable for cognitively impaired participants?**	49	79.6	20.4			
**Suitable for frail participants?**	50	94.0	6.0			
**Does the act of measuring this biomarker accelerate ageing?**	50	16.0	84.0			
**Is it clinically validated (i.e., has it been validated for use in clinical settings against set clinical standards)?**	50	94.0	6.0			
**Is it mechanistically validated (i.e., does the biomarker reflect underlying cellular and molecular mechanisms of ageing)?**	50	86.0	14.0			
**Is it generalisable (i.e., does the biomarker function across different applications, i.e., cell type, organ, system, human populations)?**	50	60.0	40.0			
**Is it precise (i.e., repeatable and reproducible)?**	50	86.0	14.0			
**Is it reliable (i.e., repeatable with minimal technical variability)?**	50	88.0	12.0			
**Are sampling and source materials easily obtained including collection, storage, and processing?**	49	93.9	6.1			
**Are complex models or software required for interpretation?**	50	10.0	90.0			
**Is it sensitive?**	50	86.0	14.0			
**Is it specific?**	49	71.4	28.6			
**Can it be blinded to participants?**	50	52.0	48.0	36	55.6	44.4
**Can it be blinded to researchers?**	50	50.0	50.0			
**Can it be blinded to data analysts?**	50	88.0	12.0			
**Does it predict functional aspects of ageing better than chronological ageing?**	48	89.6	10.4			
**Is it responsive (i.e., does it respond to accelerated or decelerated ageing)?**	48	89.6	10.4			
**Is this biomarker...**	50					
• **Minimal burden**		74.0				
• **Moderate burden**		22.0				
• **Burdensome**		4.0				
**Is this biomarker...**	50					
• **Non-invasive**		92.0				
• **Moderately invasive**		8.0				
• **Invasive**		0.0				
**Is this biomarker...**	50			37		
• **Minimal financial cost (< $10/participant)**		62.0			78.4	
• **Low financial cost ($10-50/participant)**		32.0			18.9	
• **Moderate financial cost ($51-100/participant)**		6.0			2.7	
• **High financial cost ($101-1000+ /participant)**		0.0			0.0	

eTable 14: Responses to statements for hand grip strength (HGS) across Round 2 and 3.

Hand grip strength (HGS)	Round 2			Round 3		
**Statements**	**n**	**Yes**	**No**	**n**	**Yes**	**No**
Suitable as an outcome for acute intervention studies?	56	55.4	44.6	43	69.8	30.2
Suitable as an outcome for short term intervention (< 3 months) studies?	56	75.0	25.0			
Suitable as an outcome for medium term intervention (3-6 months) studies?	56	89.3	10.7			
Suitable as an outcome for long term intervention (≥ 6 months) studies?	56	92.9	7.1			
Suitable for field settings?	56	96.4	3.6			
Suitable for cognitively impaired participants?	56	73.2	26.8			
Suitable for frail participants?	56	92.9	7.1			
Does the act of measuring this biomarker accelerate ageing?	56	25.0	75.0			
Is it clinically validated (i.e., has it been validated for use in clinical settings against set clinical standards)?	56	94.6	5.4			
Is it mechanistically validated (i.e., does the biomarker reflect underlying cellular and molecular mechanisms of ageing)?	55	74.5	25.5			
Is it generalisable (i.e., does the biomarker function across different applications, i.e., cell type, organ, system, human populations)?	56	50.0	50.0			
Is it precise (i.e., repeatable and reproducible)?	55	89.1	10.9			
Is it reliable (i.e., repeatable with minimal technical variability)?	56	91.1	8.9			
Are sampling and source materials easily obtained including collection, storage, and processing?	56	98.2	1.8			
Are complex models or software required for interpretation?	56	7.1	92.9			
Is it sensitive?	56	76.8	23.2			
Is it specific?	56	60.7	39.3	44	70.5	29.5
Can it be blinded to participants?	55	47.3	52.7	43	34.9	65.1
Can it be blinded to researchers?	55	56.4	43.6	43	62.8	37.2
Can it be blinded to data analysts?	55	89.1	10.9			
Does it predict functional aspects of ageing better than chronological ageing?	55	89.1	10.9			
Is it responsive (i.e., does it respond to accelerated or decelerated ageing)?	56	91.1	8.9			
Is this biomarker...	56					
• Minimal burden		87.5				
• Moderate burden		10.7				
• Burdensome		1.8				
Is this biomarker...	56					
• Non-invasive		96.4				
• Moderately invasive		3.6				
• Invasive		0.0				
Is this biomarker...	55					
• Minimal financial cost (< $10/participant)		81.8				
• Low financial cost ($10-50/participant)		16.4				
• Moderate financial cost ($51-100/participant)		1.8				
• High financial cost ($101-1000+ /participant)		0.0				

eTable 15: Responses to statements for Timed-Up-and-Go (TUG) across Round 2 and 3.

Timed-Up-and-Go (TUG)	Round 2			Round 3		
Statements	n	Yes	No	n	Yes	No
Suitable as an outcome for acute intervention studies?	49	55.1	44.9	37	62.2	37.8
Suitable as an outcome for short term intervention (< 3 months) studies?	49	83.7	16.3			
Suitable as an outcome for medium term intervention (3-6 months) studies?	48	95.8	4.2			
Suitable as an outcome for long term intervention (≥ 6 months) studies?	50	98.0	2.0			
Suitable for field settings?	50	94.0	6.0			
Suitable for cognitively impaired participants?	50	62.0	38.0	37	78.4	21.6
Suitable for frail participants?	50	80.0	20.0			
Does the act of measuring this biomarker accelerate ageing?	48	20.0	80.0			
Is it clinically validated (i.e., has it been validated for use in clinical settings against set clinical standards)?	49	57.1	42.9	36	91.7	8.3
Is it mechanistically validated (i.e., does the biomarker reflect underlying cellular and molecular mechanisms of ageing)?	48	57.1	42.9	37	67.6	32.4
Is it generalisable (i.e., does the biomarker function across different applications, i.e., cell type, organ, system, human populations)?	48	58.3	41.7	36	77.8	22.2
Is it precise (i.e., repeatable and reproducible)?	49	73.5	26.5			
Is it reliable (i.e., repeatable with minimal technical variability)?	48	89.6	10.4			
Are sampling and source materials easily obtained including collection, storage, and processing?	50	94.0	6.0			
Are complex models or software required for interpretation?	50	10.0	90.0			
Is it sensitive?	50	66.0	34.0	37	78.4	21.6
Is it specific?	50	60.0	40.0	37	73.0	27.0
Can it be blinded to participants?	49	44.9	55.1	37	32.4	67.6
Can it be blinded to researchers?	49	49.0	51.0	37	40.5	59.5
Can it be blinded to data analysts?	49	89.8	10.2			
Does it predict functional aspects of ageing better than chronological ageing?	48	87.5	12.5			
Is it responsive (i.e., does it respond to accelerated or decelerated ageing)?	49	89.8	10.2			
Is this biomarker...	50					
• Minimal burden		78.0				
• Moderate burden		20.0				
• Burdensome		2.0				
Is this biomarker...	50					
• Non-invasive		94.0				
• Moderately invasive		6.0				
• Invasive		0.0				
Is this biomarker...	49					
• Minimal financial cost (< $10/participant)		79.6				
• Low financial cost ($10-50/participant)		16.3				
• Moderate financial cost ($51-100/participant)		4.1				
• High financial cost ($101-1000+ /participant)		0.0				

eTable 16: Responses to statements for standing balance test (SBT) across Round 2 and 3.

Standing balance test (SBT)	Round 2			Round 3		
**Statements**	**n**	**Yes**	**No**	**n**	**Yes**	**No**
**Suitable as an outcome for acute intervention studies?**	28	53.6	46.4	23	69.6	30.4
**Suitable as an outcome for short term intervention (< 3 months) studies?**	29	79.3	20.7			
**Suitable as an outcome for medium term intervention (3-6 months) studies?**	29	93.1	6.9			
**Suitable as an outcome for long term intervention (≥ 6 months) studies?**	29	93.1	6.9			
**Suitable for field settings?**	29	96.6	3.4			
**Suitable for cognitively impaired participants?**	29	65.5	34.5	23	82.6	17.4
**Suitable for frail participants?**	29	82.8	17.2			
**Does the act of measuring this biomarker accelerate ageing?**	29	17.2	82.8			
**Is it clinically validated (i.e., has it been validated for use in clinical settings against set clinical standards)?**	28	67.9	32.1	22	90.9	9.1
**Is it mechanistically validated (i.e., does the biomarker reflect underlying cellular and molecular mechanisms of ageing)?**	29	62.1	37.9	23	82.6	17.4
**Is it generalisable (i.e., does the biomarker function across different applications, i.e., cell type, organ, system, human populations)?**	29	58.6	41.4	23	78.3	21.7
**Is it precise (i.e., repeatable and reproducible)?**	28	67.9	32.1	22	86.4	13.6
**Is it reliable (i.e., repeatable with minimal technical variability)?**	28	71.4	28.6			
**Are sampling and source materials easily obtained including collection, storage, and processing?**	29	93.1	6.9			
**Are complex models or software required for interpretation?**	29	13.8	86.2			
**Is it sensitive?**	28	64.3	35.7	21	85.7	14.3
**Is it specific?**	28	57.1	42.9	22	59.1	40.9
**Can it be blinded to participants?**	28	42.9	57.1	22	31.8	68.2
**Can it be blinded to researchers?**	28	53.6	46.4	23	65.2	34.8
**Can it be blinded to data analysts?**	28	89.3	10.7			
**Does it predict functional aspects of ageing better than chronological ageing?**	28	82.1	17.9			
**Is it responsive (i.e., does it respond to accelerated or decelerated ageing)?**	27	92.6	7.4			
**Is this biomarker...**	29					
• **Minimal burden**		89.7				
• **Moderate burden**		10.3				
• **Burdensome**		0.0				
**Is this biomarker...**	29					
• **Non-invasive**		93.1				
• **Moderately invasive**		6.9				
• **Invasive**		0.0				
**Is this biomarker...**	29					
• **Minimal financial cost (< $10/participant)**		75.9				
• **Low financial cost ($10-50/participant)**		24.1				
• **Moderate financial cost ($51-100/participant)**		0.0				
• **High financial cost ($101-1000+ /participant)**		0.0				

eTable 17: Responses to statements for gait speed across Round 2 and 3.

Gait speed	Round 2			Round 3		
Statements	n	Yes	No	n	Yes	No
Suitable as an outcome for acute intervention studies?	42	85.7	14.3			
Suitable as an outcome for short term intervention (< 3 months) studies?	41	90.2	9.8			
Suitable as an outcome for medium term intervention (3-6 months) studies?	43	88.4	11.6			
Suitable as an outcome for long term intervention (≥ 6 months) studies?	43	97.7	2.3			
Suitable for field settings?	43	95.3	4.7			
Suitable for cognitively impaired participants?	42	78.6	21.4			
Suitable for frail participants?	43	93.0	7.0			
Does the act of measuring this biomarker accelerate ageing?	43	18.6	81.4			
Is it clinically validated (i.e., has it been validated for use in clinical settings against set clinical standards)?	42	90.5	9.5			
Is it mechanistically validated (i.e., does the biomarker reflect underlying cellular and molecular mechanisms of ageing)?	42	73.8	26.2			
Is it generalisable (i.e., does the biomarker function across different applications, i.e., cell type, organ, system, human populations)?	43	65.1	34.9	32	90.6	9.4
Is it precise (i.e., repeatable and reproducible)?	43	79.1	20.9			
Is it reliable (i.e., repeatable with minimal technical variability)?	42	81.0	19.0			
Are sampling and source materials easily obtained including collection, storage, and processing?	43	100.0	0.0			
Are complex models or software required for interpretation?	43	11.6	88.4			
Is it sensitive?	43	65.1	34.9	31	80.6	19.4
Is it specific?	42	59.5	40.5	32	75.0	25.0
Can it be blinded to participants?	42	38.1	61.9	32	31.3	68.8
Can it be blinded to researchers?	41	53.7	46.3	32	59.4	40.6
Can it be blinded to data analysts?	42	90.5	9.5			
Does it predict functional aspects of ageing better than chronological ageing?	42	85.7	14.3			
Is it responsive (i.e., does it respond to accelerated or decelerated ageing)?	41	90.2	9.8			
Is this biomarker...	43					
• Minimal burden		88.4				
• Moderate burden		11.6				
• Burdensome		0.0				
Is this biomarker...	43					
• Non-invasive		97.7				
• Moderately invasive		2.3				
• Invasive		0.0				
Is this biomarker...	43					
• Minimal financial cost (< $10/participant)		81.4				
• Low financial cost ($10-50/participant)		18.6				
• Moderate financial cost ($51-100/participant)		0.0				
• High financial cost ($101-1000+ /participant)		0.0				

eTable 18: Responses to statements for frailty index across Round 2 and 3.

Frailty index	Round 2			Round 3		
Statements	n	Yes	No	n	Yes	No
Suitable as an outcome for acute intervention studies?	36	30.6	69.4	24	8.3	91.7
Suitable as an outcome for short term intervention (< 3 months) studies?	36	52.8	47.2	24	54.2	45.8
Suitable as an outcome for medium term intervention (3-6 months) studies?	36	75.0	25.0			
Suitable as an outcome for long term intervention (≥ 6 months) studies?	36	94.4	5.6			
Suitable for field settings?	36	88.9	11.1			
Suitable for cognitively impaired participants?	36	86.1	13.9			
Suitable for frail participants?	36	100.0	0.0			
Does the act of measuring this biomarker accelerate ageing?	36	13.9	86.1			
Is it clinically validated (i.e., has it been validated for use in clinical settings against set clinical standards)?	35	91.4	8.6			
Is it mechanistically validated (i.e., does the biomarker reflect underlying cellular and molecular mechanisms of ageing)?	36	69.4	30.6	24	79.2	20.8
Is it generalisable (i.e., does the biomarker function across different applications, i.e., cell type, organ, system, human populations)?	36	72.2	27.8			
Is it precise (i.e., repeatable and reproducible)?	35	62.9	37.1	24	70.8	29.2
Is it reliable (i.e., repeatable with minimal technical variability)?	35	80.0	20.0			
Are sampling and source materials easily obtained including collection, storage, and processing?	36	91.7	8.3			
Are complex models or software required for interpretation?	36	16.7	83.3			
Is it sensitive?	36	66.7	33.3	24	70.8	29.2
Is it specific?	36	41.7	58.3	23	21.7	78.3
Can it be blinded to participants?	36	86.1	13.9			
Can it be blinded to researchers?	36	63.9	36.1	24	83.3	16.7
Can it be blinded to data analysts?	36	94.4	5.6			
Does it predict functional aspects of ageing better than chronological ageing?	36	86.1	13.9			
Is it responsive (i.e., does it respond to accelerated or decelerated ageing)?	35	88.6	11.4			
Is this biomarker...	36			24		
• Minimal burden		69.4			91.7	
• Moderate burden		27.8			8.3	
• Burdensome		2.8			0.0	
Is this biomarker...	36					
• Non-invasive		94.4				
• Moderately invasive		5.6				
• Invasive		0.0				
Is this biomarker...	35			23		
• Minimal financial cost (< $10/participant)		62.9			78.3	
• Low financial cost ($10-50/participant)		34.3			21.7	
• Moderate financial cost ($51-100/participant)		2.9			0.0	
• High financial cost ($101-1000+ /participant)		0.0			0.0	

eTable 19: Responses to statements for cognitive health across Round 2 and 3.

Cognitive health	Round 2			Round 3		
**Statements**	**n**	**Yes**	**No**	**n**	**Yes**	**No**
**Suitable as an outcome for acute intervention studies?**	33	54.5	45.5	23	65.2	34.8
**Suitable as an outcome for short term intervention (< 3 months) studies?**	33	69.7	30.3	23	78.3	21.7
**Suitable as an outcome for medium term intervention (3-6 months) studies?**	32	78.1	21.9			
**Suitable as an outcome for long term intervention (≥ 6 months) studies?**	33	97.0	3.0			
**Suitable for field settings?**	33	97.0	3.0			
**Suitable for cognitively impaired participants?**	33	90.9	9.1			
**Suitable for frail participants?**	33	97.0	3.0			
**Does the act of measuring this biomarker accelerate ageing?**	33	21.2	78.8			
**Is it clinically validated (i.e., has it been validated for use in clinical settings against set clinical standards)?**	32	90.6	9.4			
**Is it mechanistically validated (i.e., does the biomarker reflect underlying cellular and molecular mechanisms of ageing)?**	32	75.0	25.0			
**Is it generalisable (i.e., does the biomarker function across different applications, i.e., cell type, organ, system, human populations)?**	33	66.7	33.3	23	82.6	17.4
**Is it precise (i.e., repeatable and reproducible)?**	33	84.8	15.2			
**Is it reliable (i.e., repeatable with minimal technical variability)?**	33	81.8	18.2			
**Are sampling and source materials easily obtained including collection, storage, and processing?**	33	97.0	3.0			
**Are complex models or software required for interpretation?**	33	33.3	66.7	23	21.7	78.3
**Is it sensitive?**	33	69.7	30.3	23	82.6	17.4
**Is it specific?**	33	60.6	39.4	23	69.6	30.4
**Can it be blinded to participants?**	32	46.9	53.1	23	30.4	69.6
**Can it be blinded to researchers?**	32	53.1	46.9	23	60.9	39.1
**Can it be blinded to data analysts?**	32	93.8	6.3			
**Does it predict functional aspects of ageing better than chronological ageing?**	31	87.1	12.9			
**Is it responsive (i.e., does it respond to accelerated or decelerated ageing)?**	30	86.7	13.3			
**Is this biomarker...**	33			23		
• **Minimal burden**		57.6			60.9	
• **Moderate burden**		33.3			34.8	
• **Burdensome**		9.1			4.3	
**Is this biomarker...**	32					
• **Non-invasive**		87.5				
• **Moderately invasive**		12.5				
• **Invasive**		0.0				
**Is this biomarker...**	33					
• **Minimal financial cost (< $10/participant)**		45.5				
• **Low financial cost ($10-50/participant)**		30.3				
• **Moderate financial cost ($51-100/participant)**		18.2				
• **High financial cost ($101-1000+ /participant)**		6.1				

eTable 20: Responses to statements for blood pressure across Round 2 and 3.

Blood pressure	Round 2			Round 3		
Statements	n	Yes	No	n	Yes	No
Suitable as an outcome for acute intervention studies?	42	61.9	38.1	31	93.5	6.5
Suitable as an outcome for short term intervention (< 3 months) studies?	42	71.4	28.6			
Suitable as an outcome for medium term intervention (3-6 months) studies?	42	81.0	19.0			
Suitable as an outcome for long term intervention (≥ 6 months) studies?	42	81.0	19.0			
Suitable for field settings?	42	90.5	9.5			
Suitable for cognitively impaired participants?	42	92.9	7.1			
Suitable for frail participants?	42	92.9	7.1			
Does the act of measuring this biomarker accelerate ageing?	42	11.9	88.1			
Is it clinically validated (i.e., has it been validated for use in clinical settings against set clinical standards)?	41	92.7	7.3			
Is it mechanistically validated (i.e., does the biomarker reflect underlying cellular and molecular mechanisms of ageing)?	40	55.0	45.0	31	80.6	19.4
Is it generalisable (i.e., does the biomarker function across different applications, i.e., cell type, organ, system, human populations)?	42	47.6	52.4	30	56.7	43.3
Is it precise (i.e., repeatable and reproducible)?	41	85.4	14.6			
Is it reliable (i.e., repeatable with minimal technical variability)?	41	82.9	17.1			
Are sampling and source materials easily obtained including collection, storage, and processing?	41	95.1	4.9			
Are complex models or software required for interpretation?	41	2.4	97.6			
Is it sensitive?	42	66.7	33.3	30	76.7	23.3
Is it specific?	42	54.8	45.2	29	62.1	37.9
Can it be blinded to participants?	41	63.4	36.6	30	80.0	20.0
Can it be blinded to researchers?	41	53.7	46.3	30	70.0	30.0
Can it be blinded to data analysts?	40	90.0	10.0			
Does it predict functional aspects of ageing better than chronological ageing?	41	36.6	63.4	29	41.4	58.6
Is it responsive (i.e., does it respond to accelerated or decelerated ageing)?	40	52.5	47.5	29	69.0	31.0
Is this biomarker...	42					
• Minimal burden		90.5				
• Moderate burden		7.1				
• Burdensome		2.4				
Is this biomarker...	42					
• Non-invasive		95.2				
• Moderately invasive		4.8				
• Invasive		0.0				
Is this biomarker...	42					
• Minimal financial cost (< $10/participant)		90.5				
• Low financial cost ($10-50/participant)		9.5				
• Moderate financial cost ($51-100/participant)		0.0				
• High financial cost ($101-1000+ /participant)		0.0				

eTable 21: Responses to statements for telomere length across Round 2 and 3.

Telomere length	Round 2			Round 3		
**Statements**	**n**	**Yes**	**No**	**n**	**Yes**	**No**
**Suitable as an outcome for acute intervention studies?**	22	22.7	77.3			
**Suitable as an outcome for short term intervention (< 3 months) studies?**	22	36.4	63.6	17	17.6	82.4
**Suitable as an outcome for medium term intervention (3-6 months) studies?**	21	38.1	61.9	17	23.5	76.5
**Suitable as an outcome for long term intervention (≥ 6 months) studies?**	22	86.4	13.6			
**Suitable for field settings?**	22	50.0	50.0			
**Suitable for cognitively impaired participants?**	22	95.5	4.5			
**Suitable for frail participants?**	22	90.9	9.1			
**Does the act of measuring this biomarker accelerate ageing?**	22	13.6	86.4			
**Is it clinically validated (i.e., has it been validated for use in clinical settings against set clinical standards)?**	21	28.6	71.4			
**Is it mechanistically validated (i.e., does the biomarker reflect underlying cellular and molecular mechanisms of ageing)?**	22	95.5	4.5			
**Is it generalisable (i.e., does the biomarker function across different applications, i.e., cell type, organ, system, human populations)?**	22	77.3	22.7			
**Is it precise (i.e., repeatable and reproducible)?**	22	54.5	45.5	17	47.1	52.9
**Is it reliable (i.e., repeatable with minimal technical variability)?**	22	68.2	31.8	17	64.7	35.3
**Are sampling and source materials easily obtained including collection, storage, and processing?**	22	86.4	13.6			
**Are complex models or software required for interpretation?**	22	50.0	50.0			
**Is it sensitive?**	22	40.9	59.1	17	35.3	64.7
**Is it specific?**	22	40.9	59.1	17	29.4	70.6
**Can it be blinded to participants?**	22	100.0	0.0			
**Can it be blinded to researchers?**	21	100.0	0.0			
**Can it be blinded to data analysts?**	22	100.0	0.0			
**Does it predict functional aspects of ageing better than chronological ageing?**	21	57.1	42.9	16	75.0	25.0
**Is it responsive (i.e., does it respond to accelerated or decelerated ageing)?**	22	90.9	9.1			
**Is this biomarker...**	22					
• **Minimal burden**		50.0				
• **Moderate burden**		50.0				
• **Burdensome**		0.0				
**Is this biomarker...**	22					
• **Non-invasive**		18.2				
• **Moderately invasive**		77.3				
• **Invasive**		4.5				
**Is this biomarker...**	22					
• **Minimal financial cost (< $10/participant)**		4.5				
• **Low financial cost ($10-50/participant)**		50.0				
• **Moderate financial cost ($51-100/participant)**		40.9				
• **High financial cost ($101-1000+ /participant)**		4.5				

eTable 22: Responses to statements for DNA methylation across Round 2 and 3 (merged with epigenetic clocks).

DNA methylation	Round 2			Round 3		
Statements	n	Yes	No	n	Yes	No
Suitable as an outcome for acute intervention studies?	25	48.0	52.0	18	33.3	66.7
Suitable as an outcome for short term intervention (< 3 months) studies?	25	52.0	48.0	18	55.6	44.4
Suitable as an outcome for medium term intervention (3-6 months) studies?	25	76.0	24.0			
Suitable as an outcome for long term intervention (≥ 6 months) studies?	25	92.0	8.0			
Suitable for field settings?	25	80.0	20.0			
Suitable for cognitively impaired participants?	25	96.0	4.0			
Suitable for frail participants?	25	96.0	4.0			
Does the act of measuring this biomarker accelerate ageing?	25	32.0	68.0	18	27.8	72.2
Is it clinically validated (i.e., has it been validated for use in clinical settings against set clinical standards)?	25	36.0	64.0	18	16.7	83.3
Is it mechanistically validated (i.e., does the biomarker reflect underlying cellular and molecular mechanisms of ageing)?	25	80.0	20.0			
Is it generalisable (i.e., does the biomarker function across different applications, i.e., cell type, organ, system, human populations)?	25	84.0	16.0			
Is it precise (i.e., repeatable and reproducible)?	25	72.0	28.0			
Is it reliable (i.e., repeatable with minimal technical variability)?	25	88.0	12.0			
Are sampling and source materials easily obtained including collection, storage, and processing?	25	88.0	12.0			
Are complex models or software required for interpretation?	24	75.0	25.0			
Is it sensitive?	25	68.0	32.0	18	83.3	16.7
Is it specific?	25	36.0	64.0	17	35.3	64.7
Can it be blinded to participants?	25	100.0	0.0			
Can it be blinded to researchers?	25	96.0	4.0			
Can it be blinded to data analysts?	25	100.0	0.0			
Does it predict functional aspects of ageing better than chronological ageing?	24	79.2	20.8			
Is it responsive (i.e., does it respond to accelerated or decelerated ageing)?	24	91.7	8.3			
Is this biomarker...	25			16		
• Minimal burden		60.0			62.5	
• Moderate burden		28.0			31.3	
• Burdensome		12.0			6.3	
Is this biomarker...	25			18		
• Non-invasive		32.0			22.2	
• Moderately invasive		68.0			77.8	
• Invasive		0.0			0.0	
Is this biomarker...	25			18		
• Minimal financial cost (< $10/participant)		4.0			0.0	
• Low financial cost ($10-50/participant)		16.0			5.6	
• Moderate financial cost ($51-100/participant)		16.0			11.1	
• High financial cost ($101-1000+ /participant)		64.0			83.3	

eTable 23: Responses to statements for epigenetic clocks across Round 2 and 3 (merged with DNA methylation).

Epigenetic clocks	Round 2		
**Statements**	**n**	**Yes**	**No**
Suitable as an outcome for acute intervention studies?	20	45.0	55.0
Suitable as an outcome for short term intervention (< 3 months) studies?	20	45.0	55.0
Suitable as an outcome for medium term intervention (3-6 months) studies?	20	70.0	30.0
Suitable as an outcome for long term intervention (≥ 6 months) studies?	20	85.0	15.0
Suitable for field settings?	20	85.0	15.0
Suitable for cognitively impaired participants?	20	80.0	20.0
Suitable for frail participants?	20	95.0	5.0
Does the act of measuring this biomarker accelerate ageing?	20	30.0	70.0
Is it clinically validated (i.e., has it been validated for use in clinical settings against set clinical standards)?	20	40.0	60.0
Is it mechanistically validated (i.e., does the biomarker reflect underlying cellular and molecular mechanisms of ageing)?	20	85.0	15.0
Is it generalisable (i.e., does the biomarker function across different applications, i.e., cell type, organ, system, human populations)?	20	90.0	10.0
Is it precise (i.e., repeatable and reproducible)?	20	75.0	25.0
Is it reliable (i.e., repeatable with minimal technical variability)?	20	80.0	20.0
Are sampling and source materials easily obtained including collection, storage and processing?	20	80.0	20.0
Are complex models or software required for interpretation?	20	95.0	5.0
Is it sensitive?	20	65.0	35.0
Is it specific?	20	45.0	55.0
Can it be blinded to participants?	20	100.0	0.0
Can it be blinded to researchers?	20	95.0	5.0
Can it be blinded to data analysts?	20	100.0	0.0
Does it predict functional aspects of ageing better than chronological ageing?	19	84.2	15.8
Is it responsive (i.e., does it respond to accelerated or decelerated ageing)?	19	94.7	5.3
Is this biomarker...	20		
• Minimal burden		45.0	
• Moderate burden		50.0	
• Burdensome		5.0	
Is this biomarker...	20		
• Non-invasive		20.0	
• Moderately invasive		75.0	
• Invasive		5.0	
Is this biomarker...	20		
• Minimal financial cost (< $10/participant)		10.0	
• Low financial cost ($10-50/participant)		20.0	
• Moderate financial cost ($51-100/participant)		70.0	
• High financial cost ($101-1000+ /participant)		0.0	
